# Amine-Regulated pri-SMTP Oxidation in SMTP Biosynthesis in *Stachybotrys*: Possible Implication in Nitrogen Acquisition

**DOI:** 10.3390/jof8090975

**Published:** 2022-09-18

**Authors:** Ryota Iwama, Yu Sasano, Taichi Hiramatsu, Shinya Otake, Eriko Suzuki, Keiji Hasumi

**Affiliations:** 1 Department of Applied Biological Science, Tokyo University of Agriculture and Technology, Fuchu, Tokyo 183-8509, Japan; 2 Department of Research and Development, TMS Co., Fuchu, Tokyo 183-0055, Japan

**Keywords:** nitrogen acquisition, secondary metabolism, oxidase, SMTP, compartmentalization, biosynthesis, black mold, *Stachybotrys microspora*

## Abstract

SMTP (the name SMTP is derived from *Stachybotrys microspora* triprenyl phenol) is a family of triprenyl phenol secondary metabolites from a black mold, *Stachybotrys microspora*. Some SMTP congeners exhibit anti-inflammatory and profibrinolytic activities that, in combination, contribute to the treatment of ischemic stroke. The final step in the SMTP biosynthesis is a non-enzymatic amine conjugation with an *o*-phthalaldehyde moiety of the precursor pre-SMTP, which can form adducts with proteins and nucleic acids. Thus, pre-SMTP formation should be a precisely regulated, rate-limiting step in the SMTP biosynthesis. To address the mechanism backing this regulation, we purified a metabolite that rapidly disappeared following amine feeding, identifying a novel compound, pri-SMTP. Furthermore, an enzyme, designated as pri-SMTP oxidase, responsible for pri-SMTP conversion to pre-SMTP, was purified. The formation of pri-SMTP, which is regulated by nitrogen and carbon nutrients, occurred in particular septate mycelia. Although pri-SMTP oxidase was expressed constitutively, the consumption of pri-SMTP was accelerated only when a primary amine was fed. Thus, SMTP biosynthesis is regulated by at least three mechanisms: (i) pri-SMTP formation affected by nutrients, (ii) the compartmentalization of pri-SMTP formation/storage, and (iii) amine-regulated pri-SMTP oxidation. Amine-regulated SMTP formation (i.e., amine-capturing with pre-SMTP) may play a role in the nitrogen acquisition/assimilation strategy in *S. microspora*, since pri-SMTP synthesis occurs on non-preferred nitrogen.

## 1. Introduction

SMTP is a family of triprenyl phenol secondary metabolites produced by a black mold, *Stachybotrys microspora* (*Stachybotrys microspora* triprenyl phenol) [1,2]. Triprenyl phenols are characteristic metabolites in the genera *Stachybotrys* and *Memnoniella*. This family consists of various compounds with significant biological activities such as inhibitions of the complement system, fucosyltransferases, *myo*-inositol monophosphatase, syalyltransferases, cholesterol esterase, receptor tyrosine kinase, glucose-6-phosphate translocase, human immunodeficiency virus reverse transcriptase, and farnesyl-protein transferase, as well as the modulation of cholesteryl ester transfer among lipoproteins, endothelin-binding antagonism, the suppression of lipid accumulation in hepatocytes, and the promotion of neurite outgrowth [3]. The SMTP family compounds are unique in that some of them exhibit plasminogen modulation activity to promote physiological fibrinolysis [1,2,4]. Moreover, some SMTP congeners are inhibitory to soluble epoxide hydrolase and exhibit anti-inflammatory activities [1,5,6,7,8,9,10,11]. SMTP congeners with both activities are useful for treating ischemic stroke, which is caused by an occlusion of a cerebral artery and is exacerbated by inflammation [1]. One of the SMTP congeners is under clinical development for the treatment of acute ischemic stroke [1].

**Figure 1:**
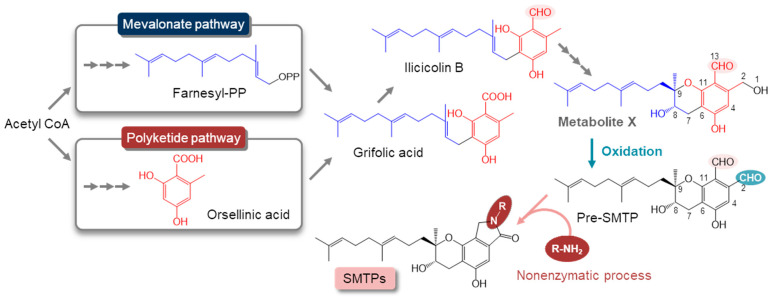
Putative pathway for the biosynthesis of SMTP. SMTP can originate from grifolic acid, which is derived from farnesyl pyrophosphate and orsellinic acid. Grifolic acid undergoes reduction to form ilicicolin B, which can derive pre-SMTP, which has an amine-reactive, aromatic ortho-dialdehyde function. A non-enzymatic reaction between pre-SMTP and a primary amine affords a variety of SMTP congeners that differ in the *N*-linked side chain. We hypothesize that pre-SMTP is formed via an enzymatic oxidation of the alcoholic function at position 1 of the speculative metabolite X.

It has been postulated that the biosynthesis of triprenyl phenols are comprised of two distinct pathways: the isoprene biosynthesis to afford farnesyl diphosphate, and the polyketide pathway to yield orsellinic acid [12]. The docking of the two metabolites forms the triprenyl phenol precursor, grifolic acid (Figure 1), which can convert into a variety of triprenyl phenol metabolites such as SMTPs/stachybotrins and phenylspirodrimanes through different cyclization mechanisms [1]. The final step in SMTP biosynthesis is a non-enzymatic reaction between pre-SMTP and a primary amine present inside the cells or in the medium (Figure 1). This reaction involves the aromatic *ortho*-dialdehyde moiety of pre-SMTP, which is highly reactive toward primary amines. Similarly, another reactive aromatic *ortho*-dialdehyde, stachybotrydial, can be involved in the biosynthesis of amine-coupled phenylspirodrimanes of vast structural diversity in other *Stachybotrys* species [1,3]. These findings suggest a role for the biosynthesis of the reactive aromatic *ortho*-dialdehydes in nitrogen acquisition in *Stachybotrys*. However, aromatic *ortho*-dialdehydes can form covalent adducts by reacting with biologically essential amines such as nucleic acids and proteins [13,14]. Thus, the formation of aromatic *ortho*-dialdehydes should be precisely regulated to avoid such unfavorable reactions, whereas the mechanism of this regulation remains unknown.

This study was aimed at unraveling the regulatory mechanism for pre-SMTP formation. To do so, we first searched for a precursor (“metabolite X” in Figure 1) to pre-SMTP. Next, we purified an enzyme responsible for pre-SMTP formation from metabolite X, thus designating metabolite X as pri-SMTP. Our results demonstrate that pre-SMTP is formed from pri-SMTP through an enzymatic oxidation by pri-SMTP oxidase. Pri-SMTP oxidase is expressed constitutively, whereas pri-SMTP consumption is accelerated only when a primary amine is available. This amine-dependent oxidation of pri-SMTP and the amine-capturing with the resulting pre-SMTP can consist of a mechanism that plays a role in nitrogen acquisition in *S. microspora*, since pri-SMTP synthesis occurs on non-preferred nitrogen.

## 2. Materials and Methods

### 2.1. Materials

*S. microspora* NBRC 30018 (formerly registered as IFO 30018), which was isolated from a fallen leaf of *Castanopsis cuspidata* var. *sieboldii*, collected at the riverside of Kuira, Iriomote island, Okinawa, Japan, was used as a producer of SMTP and its precursors. Pre-SMTP, SMTP-0, SMTP-4, and SMTP-7, used as standards for the analyses, were prepared as described previously [15,16,17,18]. The compositions of the buffers used were: buffer A, 50 mM HEPES-NaOH, pH 7.4, 1 mM EDTA, 0.6 mM sorbitol, and 2 mM 2-mercaptoethanol; buffer B, 20 mM HEPES-NaOH; buffer C, 20 mM HEPES-NaOH, pH 7.4, and 1 M NaCl; and buffer D, 200 mM sodium phosphate, pH 6.8. *N*_δ_-FITC−l-ornithine (FITC−Orn) was prepared by incubating L-ornithine with 10 molar excess of FITC, followed by purification on preparative HPLC.

### 2.2. Production and Determination of the Precursor of SMTP Biosynthesis (Metabolite X)

The seed culture medium consisted of (% in wt vol^−1^) glucose (4%), soybean meal (0.5%), peptone (0.3%), yeast extract (0.3%), and the antifoam CB442 (Nippon Oil & Fat Co., Tokyo, Japan) (0.01%), pH 5.8; and the production culture medium contained sucrose (5%), yeast extract (0.1%), KNO_3_ (0.7%; 70 mM), K_2_HPO_4_ (0.1%), MgSO_4_∙7H_2_O (0.05%), KCl (0.05%), CoCl_2_∙6H_2_O (0.00025%), FeSO_4_∙7H_2_O (0.0015%), CaCl_2_∙H_2_O (0.00065%), and CB442 (0.01%), pH 5.8. *S. microspora* NBRC 30018 was incubated at 25 °C for 4 days in a 500 mL Erlenmeyer flask containing 100 mL of the seed medium on a rotary shaker at 180 rpm. The seed culture (5 mL) was transferred to a 500 mL Erlenmeyer flask containing 100 mL of the production medium (day 0). The flask was incubated at 25 °C on a rotary shaker at 180 rpm for 96 h. Mycelium was collected from 1 mL of the culture suspension via centrifugation at 1000× *g* for 10 min, washed thrice with water, and extracted with 1 mL of acetonitrile (MeCN) for 1 h. After centrifugation, the MeCN extracts were subjected to ultra-performance liquid chromatography (UPLC) to determine the level of metabolites. Detailed conditions for the analysis are described in Section 2.4.

### 2.3. Production and Determination of SMTP-7, an SMTP Family Member That Is Produced through the Conjugation of pre-SMTP with l-ornithine

The production culture mentioned in Section 2.2 received 100 mg of L-ornithine on Day 4 of the production culture (96 h after the seed inoculation), and the incubation was continued further for 24 h. The culture received 200 mL of methanol (MeOH), and metabolites were extracted and analyzed as described in Section 2.2.

### 2.4. UPLC Analysis of the Metabolites

The following conditions were used for the analyses of the metabolites from *S. microspora*: chromatograph, Altus A-30 UPLC system (PerkinElmer, Waltham, MA, USA); column, Acquity BEH C18 1.7 µm (2.1 × 50 mm, Waters, Milford, MA, USA); elution conditions, a linear gradient of 0.1% (vol vol^−1^) formic acid (80 to 0%) in MeCN for 4 min, followed by an isocratic elution with MeCN for 2 min at 40 °C at a flow rate of 0.5 mL min^−1^; detection, UV-visible from 210 to 400 nm using a photodiode array detector equipped in the Altus A-30 system.

### 2.5. Isolation of Metabolite X (pri-SMTP)

*S. microspora* NBRC 30018 was grown as described in Section 2.2 in 29 flasks (a total of 2.9 L of cultures). On Day 4 of the production culture, each flask received 200 mL of MeOH, and the resulting extracts were filtered and concentrated under reduced pressure to yield 2.5 L of a suspension. The soluble fraction of the suspension was applied to a column (3 × 25 cm) filled with Diaion HP-20 resin (Mitsubishi Chemical, Tokyo, Japan) at a rate of 10 mL min^−1^. The column was developed with 400 mL of MeOH, and 100 mL fractions were collected. Pri-SMTP was eluted in the second and third fractions. The eluate was mixed with the insoluble fraction of the above-mentioned suspension, yielding 3.9 g of an oily residue, which was dissolved in 20 mL of MeOH and pretreated with LiChrolut RP-18 (Merck, Frankfurter, Germany). Aliquots (4 mL) of the resulting sample were fractionated into five batches via preparative HPLC under the following conditions: column, Intersil PREP-ODS (30 × 250 mm, GL Sciences, Tokyo, Japan); elution conditions, a linear gradient of 0.1% (vol vol^−1^) formic acid (50 to 0%) in MeOH for 20 min followed by an isocratic elution with MeOH for 15 min at 40 °C with a flow rate of 25 mL min^−1^; detection, UV-visible from 200 to 400 nm using a MD-2010 photodiode array detector (JASCO, Tokyo, Japan). Fractions containing pri-SMTP, which was eluted as a peak at 21.5 min, were concentrated and lyophilized, affording 200 mg of a purified material.

### 2.6. Spectroscopic Analyses of pri-SMTP

NMR spectra were acquired on an ECA-600 (JEOL, Tokyo, Japan) using dimethyl sulfoxide (DMSO)-*d*_6_ as a solvent. UV spectra were measured on a U-2910 spectrometer (Hitachi, Tokyo, Japan) in MeCN−0.1% (vol vol^−1^) formic acid aq. (7:1) or MeOH−0.1% (vol vol^−1^) formic acid aq. (21:4) at a concentration of 50 µM. A mass spectrum was obtained on a micrOTOF-Q II−ESI-Qq-TOF spectrometer (Bruker, Billerica, MA, USA) using ESI-L Low Concentration Tuning Mix (Agilent Technologies, Santa Clara, CA, USA) as the internal standards. The sample was dissolved in MeCN−0.1% (vol vol^−1^) formic acid aq. (7:1) for the mass analysis. IR spectrum was measured on a FT/IR-4100 type A spectrometer (JASCO, Tokyo, Japan) using the KBr method. A fluorescence spectrum was acquired on a F-2500 spectrometer (Hitachi, Tokyo, Japan) as a solution in MeCN−0.1% (vol vol^−1^) formic acid aq. (7:1).

### 2.7. Analysis of the Tautomerism in pri-SMTP

Pir-SMTP dissolved in MeOH at 504 µg mL^−1^ (1.3 mM) was analyzed under the following elution conditions using the UPLC system described in Section 2.4: *elution condition 1*, a linear gradient of 0.1% (vol vol^−1^) formic acid (50 to 0%) in MeOH for 4 min, followed by an isocratic elution with MeOH for 2 min at 40 °C with a flow rate of 0.5 mL min^−1^; *elution condition 2*, a linear gradient of 0.1% (vol vol^−1^) formic acid (80 to 0%) in MeCN for 4 min, followed by an isocratic elution with MeCN for 2 min at 40 °C with a flow rate of 0.5 mL min^−1^; and *elution condition 3*, an isocratic elution with 50 mM ammonium acetate (30%) in MeOH for 10 min at 40 °C at a flow rate of 0.5 mL min^−1^. The volumes of pri-SMTP solution analyzed were 2 µL for the conditions 1 and 2, and 4 µL for condition 3.

### 2.8. Preparation of Cell Extracts

On day 4 of the production culture, *S. microspora* cells were harvested via filtration. Cells were washed with water and suspended with ice-cold buffer A. The volume of buffer A used was ~1/10 of the volume of culture. The resulting cell suspension was subjected to French press disruption at 4 °C. The resulting homogenate was centrifuged at 500× *g* for 15 min at 4 °C to obtain the supernatant, which was centrifuged at 12,000× *g* for 20 min at 4 °C. The resulting supernatant was ultracentrifuged at 105,000× *g* for 120 min at 4 °C to obtain a cytosol fraction. In some experiments where noted, we used some modified conditions of the cell extract preparation to characterize the properties of an enzyme responsible for the oxidation of pri-SMTP.

### 2.9. Assay for pri-SMTP Oxidation (Cell-Free Enzyme Assay)

The oxidation of pri-SMTP to pre-SMTP was assayed using an amine-coupling reaction developed as described below. This method is based on the fact that pre-SMTP spontaneously reacts with an amine to afford particular SMTP. When we use L-phenylalanine as an amine, SMTP-4 is produced as a product. The reason for the use of this method was to reduce the formation of pri-SMTP−protein adducts, which might bias the quantification of pre-SMTP formation. The assay reaction mixture consisted of 15 µL of buffer B containing 0.66 mM pri-SMTP and 1.67 mM l-phenylalanine, and 5 µL of cell extract or fractions of the purification steps. The mixture was incubated in a glass microtube at 25 °C for 3 h in the dark. After incubation, each tube received 180 µL of MeOH and was centrifuged at 3000× *g* for 20 min. The resulting supernatant (100 µL) was removed, and a 5 µL aliquot was analyzed for pri-SMTP and SMTP-4 via UPLC using the *elution condition 1* shown in Section 2.7.

### 2.10. Purification of pri-SMTP Oxidase

The following enzyme purification was performed at 4 °C. The cytosol fraction (280 mL) obtained from 3.6 L of *S. microspora* cultures were subjected to ammonium sulfate fractionation. The fraction precipitated between 50% and 75% saturation with ammonium sulfate was dialyzed against buffer B. After centrifugation at 12,000× *g* for 20 min, the resulting supernatant was applied to anion-exchange chromatography on a Hiprep Q HP column (5 mL, GE Healthcare, Chicago, IL, USA) pre-equilibrated with buffer B at a flow rate of 0.5 mL min^−1^. The column was washed with 22 mL of buffer B and developed with a linear gradient of buffer C in buffer B (0 to 100%) for 80 min, followed by an isocratic elution with buffer C for 40 min. The fractions containing the activity to oxidize pri-SMTP were combined and subjected to another round of anion-exchange chromatography under the same elution conditions. Finally, the resulting active fractions were fractionated using size-exclusion chromatography on a TSK gel G5000PW column (7.5 × 300 mm, TOSOH, Tokyo, Japan) equilibrated with buffer D.

### 2.11. Protein and Gene Analysis

The purified enzyme preparation was subjected to SDS–polyacrylamide gel electrophoresis on a 10% gel under non-reducing conditions. The resulting protein bands were excised from the gel and digested with trypsin. The resulting peptide fragments were analyzed using nano-LC/MS/MS on a LTQ Orbitrap Velos ETD (Thermo Fisher Scientific, Waltham, MA, USA) for peptide mass finger printing analysis under the conditions shown in Table S4. Conserved domains were searched using CD-search [19]. Protein function was predicted based on the function of the conserved domain. Secondary metabolite biosynthesis genes were searched using antiSMASH fungal version (ver. 6.0.1) [20].

### 2.12. Microscopic Localization of pri-SMTP Accumulation

Pri-SMTP exhibits a weak but discriminable fluorescence around 600–660 nm when excited at 540 nm (Figure S1). This observation enabled us to analyze the intracellular localization of pri-SMTP in unfixed, unstained *S. microspora*. The observation was performed using a BZ-X710 microscope (Keyence, Osaka, Japan) equipped with a BZ-X TexasRed filter (OP-87765, with median excitation and emission wavelengths at 560 and 630 nm, respectively). In another experiment, *S. microspora* was incubated for 60 min with FITC−Orn (50 µg mL^−1^) on Day 4 of the culture to trigger the oxidation of pri-SMTP and to label the resulting pre-SMTP to afford SMTP-48 [1], a fluorescent SMTP analog that could be visualized using a BZ-X GFP filter (OP-87763, with median excitation and emission wavelengths at 470 and 525 nm, respectively; Keyence). In these experiments, cells were washed three times with saline before the microscopic examination. In an experiment to confirm specificity of the fluorescence localization, excess amounts of L-ornithine (1 mg mL^−1^) were added to the culture 3 and 1 h before the FITC−Orn addition, and cells were washed with saline containing 1 mg mL^−1^ of l-ornithine.

## 3. Results and Discussion

### 3.1. Metabolite X Accumulates in the Amine-Restricted Culture of S. microspora

**Figure 2:**
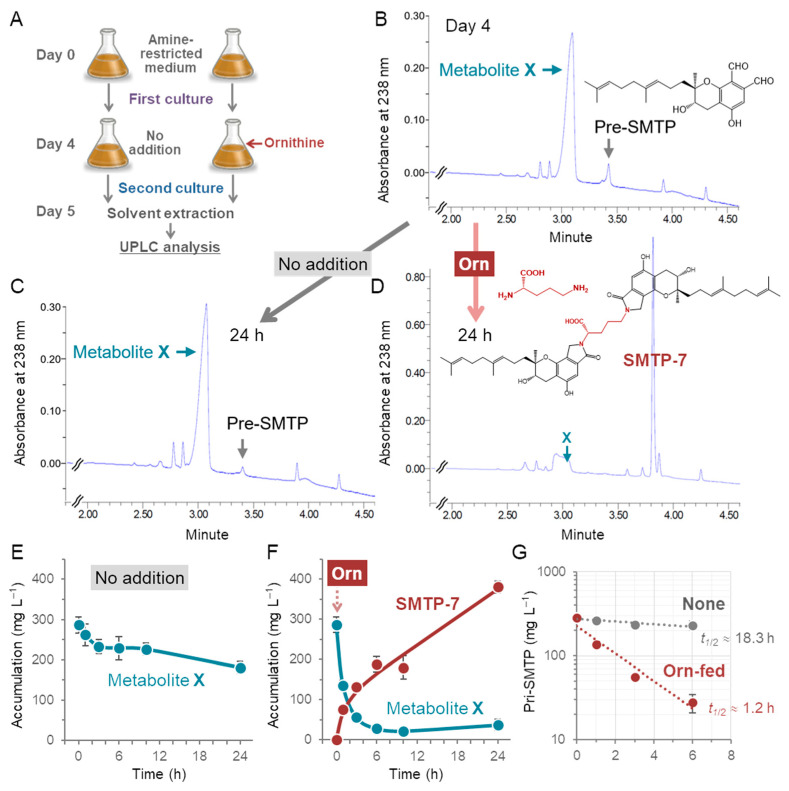
Identification of metabolites that accumulate in the amine-restricted culture and disappear following the amine feeding. (**A**) *S. microspora* was grown in the amine-restricted medium for 4 days and received L-ornithine or none on Day 4. After incubation for the indicated times, the cultures were extracted with MeCN, and the resulting extracts were analyzed using UPLC. (**B**) Before the amine feeding (Day 4), pre-SMTP and metabolite X accumulated in the culture. (**C**) These metabolites remained on Day 5 without the amine feeding on Day 4. (**D**) These metabolites were drastically consumed when L-ornithine was fed on Day 4. Instead, SMTP-7 accumulated after the L-ornithine feeding. (**E)** Time course of the changes in the levels of metabolite X without amine feeding. (**F**) Time course of the changes in the levels of SMTP-7 after L-ornithine feeding. (**G**) The rate of the decay of priSMTP after feeding of L-ornithine or none. Metabolite X persisted without amine feeding, but it was rapidly cleared following the L-ornithine feeding, accompanying a concomitant accumulation of SMTP-7. The half disappearance time (*t*_1/2_) for metabolite X was estimated to be ~1.2 h for the l-ornithine-fed culture, compared to ~18 h for the non-fed culture. Each value represents the mean ± SD (error bar) from triplicate cultures.

Typically, SMTPs are produced using a two-stage culture of *S. microspora*, where cells are first grown for 4 days in an amine-restricted medium supported by non-preferred nitrogen sources, followed by a cultivation after the feeding of a desired amine that can be incorporated into the *N*-linked side chain moiety of particular SMTP [1] (Figure 1A). Before the amine feeding, some metabolites including pre-SMTP and large amounts of unidentified metabolite (metabolite X) accumulated in the culture (Figure 2B). The level of metabolite X was rather constant unless amine was fed to the culture on Day 4 (Figure 2C,E). Metabolite X largely disappeared when the culture received L-ornithine, and SMTP-7 accumulated 24 h after the amine feeding (Figure 2D,F). Half of the metabolite X disappeared at 1.2 h after the L-ornithine addition, compared to 18.3 h without amine feeding (Figure 2G). Thus, it appears that the addition of L-ornithine triggers metabolite X conversion to pre-SMTP, which in turn couples with L-ornithine, affording SMTP-7. Similarly, other amines such as NH_4_Cl and 4-hydroxy-d-phenylglycine promote metabolite X conversion and the formation of particular SMTPs, SMTP-0, and SMTP-44D.

### 3.2. Isolation and the Structure of Metabolite X (pri-SMTP)

**Figure 3:**
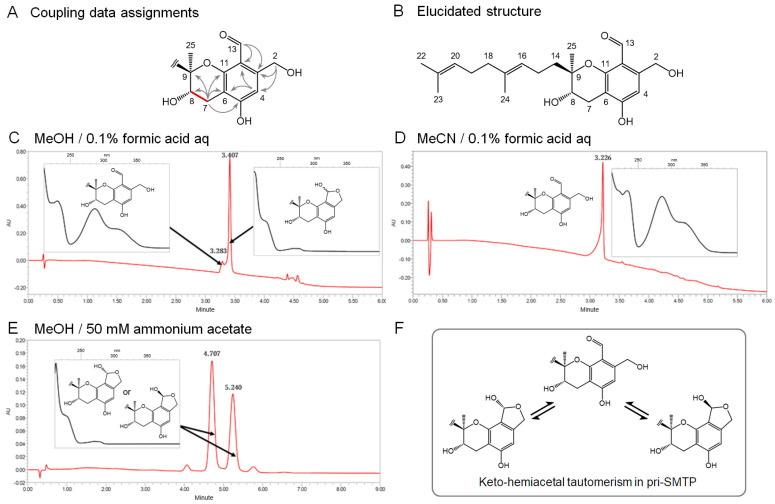
The elucidation of the structure of pri-SMTP and its keto-hemiacetal tautomerism. (**A**) Correlations observed in the ^1^H−^1^H COSY (red bond) and HMBC spectra (arrows). (**B**) The elucidated structure of the aldehyde form of pri-SMTP. (**C**) The hemiacetal form of pri-SMTP predominates over the aldehyde form in the solvent system of MeOH/0.1% formic acid. (**D**) Pri-SMTP solely exists as the aldehyde form in the solvent system of MeCN/0.1% formic acid. (**E**) The epimers of the hemiacetal form of pri-SMTP can be separable in the solvent system of MeOH/50 mM ammonium acetate. (**F**) The summary of the observed tautomerism in pri-SMTP.

To confirm the precursor–product relationship between metabolite X and pre-SMTP, we first isolated metabolite X and elucidated its structure. A solvent extraction followed by hydrophobic-interaction chromatographic fractionation and reverse phase preparative HPLC afforded 200 mg of purified metabolite X from 2.9 L of culture (see Section 2.4 for details). The structure of metabolite X was elucidated based on the combination of the spectroscopic analyses (Figure 3). The NMR data for metabolite X were mostly comparable to those for pre-SMTP [18] (Table S1). The prominent differences were: (i) the lack of the carbonyl signal at position 2 in pre-SMTP (Figure S1B), and (ii) the presence of oxymethylene signals at position 2 in metabolite X (δ_C_ 61.4 and δ_H_ 4.68) (Table S1; Figure S1). All of the physicochemical properties (Table S2) and NMR results including ^1^H−^1^H correlation spectroscopy (COSY, Figure S2A), ^1^H-detected multi-bond heteronuclear multiple quantum coherence (HMBC, Figure S2B), and ^1^H-detected multiple quantum coherence (HMQC, Figure S2C) supported the bond connection depicted in Figure 3A, as well as the proposed structure shown in Figure 3B. Along with the findings described in Section 3.1, this structure supports that metabolite X can be a direct precursor of pre-SMTP. Therefore, we designated metabolite X as pri-SMTP.

### 3.3. Tautomerism in pri-SMTP

Pri-SMTP has an aldehyde and a hydroxylmethyl function at the vicinal position, which can form a hemiacetal structure (Figure 3). Indeed, we found that pri-SMTP exhibited multiple patterns of elution and UV-visible absorption in analytical UPLC. When analyzed under *elution condition 1* (see Section 2.7), pri-SMTP mostly appeared as the hemiacetal form, along with a minor fraction corresponding to the aldehydeform (Figure 3C). The discrimination between the two tautomers was based on the UV-visible spectrum (insets in Figure 3). Under *elution condition 2*, pri-SMTP predominantly existed as the aldehyde form (Figure 3D). Two regioisomeric forms of the hemiacetal form of pri-SMTP were observed under elution condition 3 (Figure 3E). Thus, pri-SMTP exhibits two tautomeric forms, and one of the tautomers exists as two regioisomers (Figure 3F), giving rise to complicated separations in the HPLC analyses. The clarification of the elusive properties of pri-SMTP enabled us to characterize the enzymatic conversion of pri-SMTP.

### 3.4. Enzymatic Oxidation of pri-SMTP

**Figure 4:**
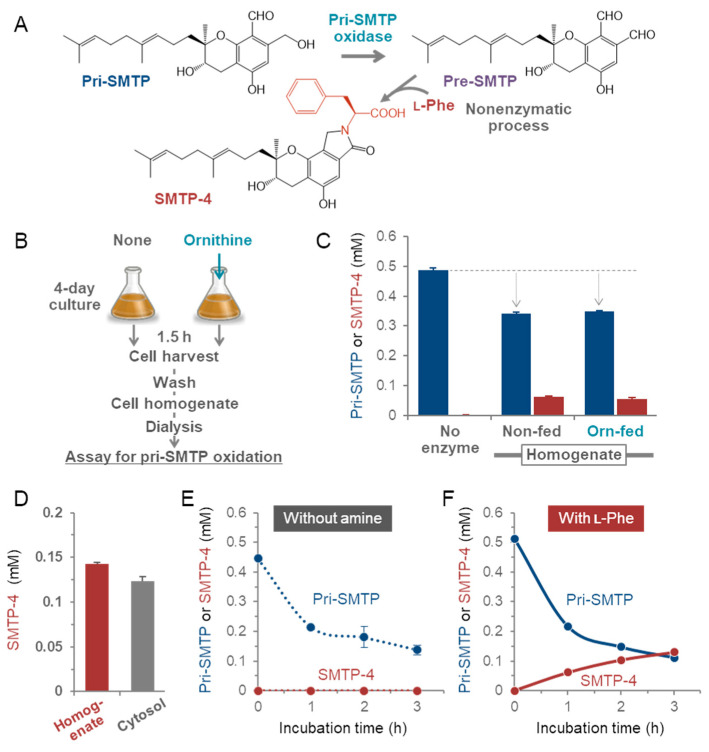
Detection of pri-SMTP oxidation in a cell-free assay. (**A**) The scheme for the cell-free assay system. The oxidation of pri-SMTP affords pre-SMTP, which is difficult to be detected in enzyme assays, since it may form adducts by reacting with amino groups in proteins present in the enzyme preparations. The inclusion of L-phenylalanine in the system facilitates the formation of SMTP-4, a product of the reaction between pre-SMTP and L-phenylalanine, thus enabling the assay for pri-SMTP oxidation by measuring the decrease in pri-SMTP and the concomitant formation of SMTP-4. (**B**) Preparation of cell homogenates. On Day 4 of the culture of *S. microspora*, L-ornithine or none was added, and culture was continued for another 1.5 h. Subsequently, cells were harvested, washed, and disrupted to prepare cell homogenates, which was then dialyzed to remove small-molecule amine compounds. An assay for pri-SMTP oxidation was performed using the dialysate. (**C**) Pri-SMTP oxidation proceeds depending on the cell homogenates. The oxidation occurs equally, whether cells have been treated or not treated with L-ornithine, demonstrating that the pri-SMTP oxidation activity is constitutively expressed, and is not induced by amine treatment. (**D**) Nearly all of the pri-SMTP oxidation activity exists in the cytosolic fraction. (**E**,**F**) Pri-SMTP oxidation proceeds without amine in the assay system, and SMTP is formed when an amine is included in the system. Each value represents the mean + SD (error bar) from triplicate determinations.

The pri-SMTP conversion to pre-SMTP in a cell-free system was assessed using an amine-coupling reaction developed as described in Section 2.9. In this assay, the pre-SMTP formed is converted to SMTP-4 with the involvement of L-phenylalanine: SMTP-4 formation occurs concomitantly with the generation of pre-SMTP (Figure 4A). The cell-free extract prepared from *S. microspora* grown in the amine-restricted medium for 4 days (Figure 4B) exhibited a significant degree of activity for converting pri-SMTP to SMTP-4 (Figure 4C). The L-ornithine-treated cells had an activity level comparable to that of the non-treated cells (Figure 4C). The enzymatic activity, which did not require any cofactors such as NAD(P)^+^ and NAD(P)H, was associated with the cytosol fraction (Figure 4D), suggesting the oxidase-like properties of the enzyme responsible. The consumption of pri-SMTP proceeded in the absence of the exogenous amine (Figure 4E) at a rate comparable to that which occurred in its presence (Figure 4F), contrasting with the result of how robust pri-SMTP consumption occurs in the culture only when exogenous amine is available (Figure 2G).

Taken together, these results can be interpreted as showing that: (i) pri-SMTP conversion to pre-SMTP is mediated by an oxidase-like enzyme that requires no oxidoreductive cofactors; (ii) this enzyme is constitutively expressed and is not induced by an amine (Figure 4C); (iii) the pri-SMTP consumption requires no amine compound in the enzyme assay (Figure 4E); (iv) the pri-SMTP consumption in the culture, on the other hand, requires an amine compound (Figure 4E); and (v) this discrepancy suggests a mechanism that enables amine-dependent pri-SMTP conversion in the culture.

### 3.5. Purification of the Enzyme Responsible for the Oxidation of pri-SMTP

**Figure 5:**
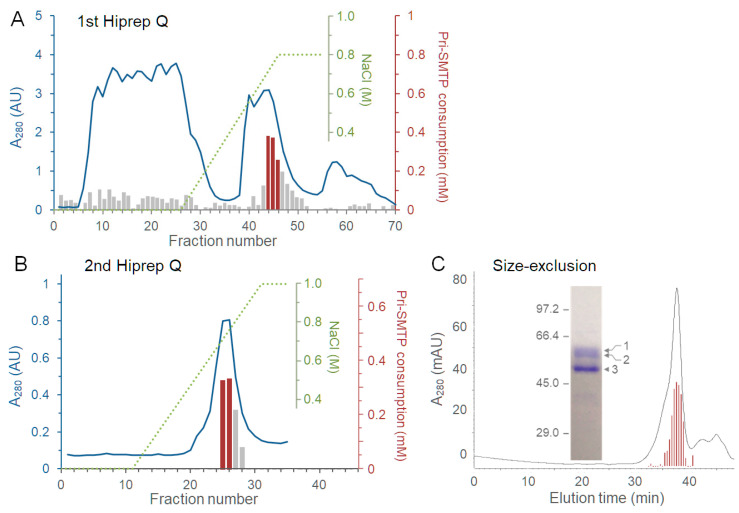
Purification of pri-SMTP oxidase. The cytosol fraction of *S. microspora* homogenate was subjected to ammonium sulfate fractionation. The precipitates at 75% saturation were dialyzed and fractionated via anion-exchange chromatography on Hiprep Q (**A**). The fractions containing pri-SMTP oxidase activity (red bars) were further fractionated via repeated chromatography on Hiprep Q (**B**). The peak fractions (red bars) were analyzed using gel permeation chromatography (**C**). The SDS-PAGE analysis of the peak fraction demonstrated one major band at 50.7 kDa, and two minor bands at 57.0 and 59.5 kDa (inset in panel (**C**)). The major protein was assigned to a putative oxidase using the peptide mass fingerprinting method (see Figure S3, Tables S4 and S5 for details), whereas the minor proteins at 57.0 and 59.5 kDa were allocated to a putative α/β-hydrolase and a putative zinc peptidase (orthologous to S40285_05629 and S40285_05629 proteins in *S. chlorohalonata* IBT 40285, respectively). In the chromatograms, solid lines, dotted lines, and bars represent the absorption at 280 nm, NaCl concentration, and pri-SMTP oxidase activity, respectively.

The enzyme responsible for the oxidative conversion of pri-SMTP to pre-SMTP, designated pri-SMTP oxidase, was purified from the cytosol fraction. Ammonium sulfate fractionation, followed by two rounds of anion-exchange chromatography, as well as gel-permeation chromatography, afforded a preparation consisting of one major protein at 50.7 kDa and two minor proteins at 57.0 and 59.5 kDa (Figure 5A–C; Table S3). The peptide mass fingerprinting analysis of peptides derived from the 50.7 kDa protein identified it as an FAD-linked oxidase, using a database for *S. chlorohalonata* IBT 40285 genomic DNA sequences [21] (Figure S3, Tables S4 and S5). This enzyme contains a glycolate oxidase subunit D superfamily domain and a berberine/berberine-like superfamily domain (Figure S4A). In the *S. chlorohalonata* IBT 4028 genome, an orthologue of pri-SMTP oxidase (pri-SMTP oxidase-like) is located in scaffold1638, where no genes for secondary metabolism are found (Figure S4). A gene cluster possibly involved in the biosynthesis of ilicicolin B and SMTP-like compounds was found in scaffold432 in the *S. chlorohalonata* IBT 4028 genome (Figure S5). Thus, the pri-SMTP oxidase-like gene locates outside of the SMTP biosynthesis gene cluster in *S. chlorohalonata* IBT 4028, whereas the pri-SMTP oxidase gene localization in *S. microspora* remains to be investigated. 

### 3.6. Regulation of the Biosynthesis of SMTP

**Figure 6:**
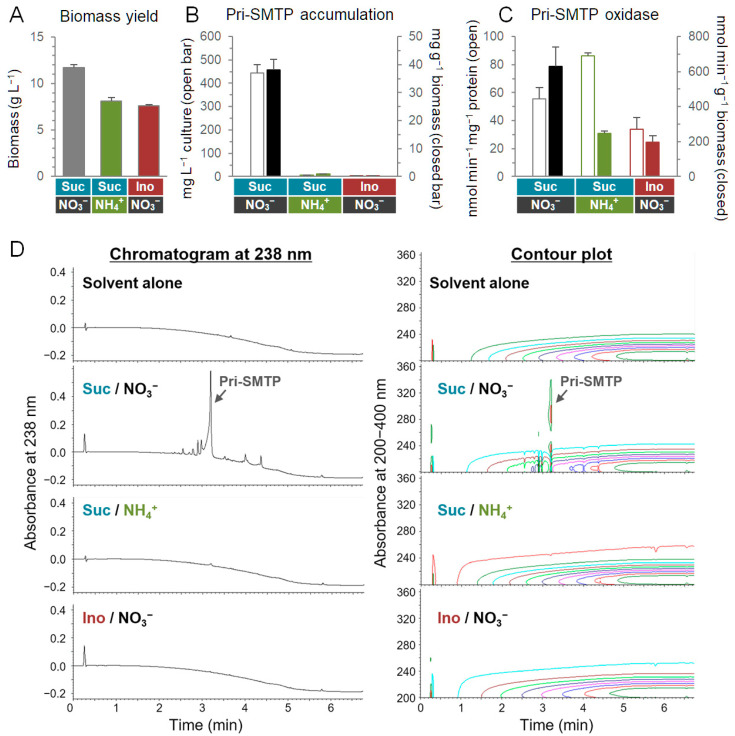
Nutrient regulation of the biosynthesis of pri-SMTP. Biomass yield (**A**), pri-SMTP accumulation (**B**), and pri-SMTP oxidase activity (**C**) in *S. microspora* grown on different nutrients. The standard medium (Suc/NO_3_^−^) consisted of sucrose (5%, wt vol^−1^) and KNO_3_ (70 mM) as the major carbon and nitrogen sources, respectively. Suc/NH_4_^+^ medium contained 70 mM NH_4_Cl in place of KNO_3_, and sucrose was replaced by *myo*-inositol (5%, wt vol^−1^) in Ino/KNO_3_ medium. Pri-SMTP oxidase activity was obtained by measuring the consumption of pri-SMTP. Each value represents the mean + SD (error bar) from triplicate cultures. (**D**) Chromatogram at 238 nm and contour plot of the UPLC analysis of the MeCN extract from *S. microspora* grown on different nutrients. The solvent system shown in Section 2.4 was used. The contour plots suggested that there was a little amount of metabolites (including those other than pri-SMTP) in the Suc/NH_4_^+^ and Ino/KNO_3_ cultures.

Typically, the production of SMTP is achieved in *S. microspora* cultures where nitrogen sources are limited, particularly on non-preferred nitrogen sources [1]. We found that the biosynthesis of SMTP was regulated by both nitrogen and carbon sources, while supporting biomass yield (Figure 6A). Sucrose-supported pri-SMTP production (Figure 6B,D) occurred in a medium consisting of the non-preferred nitrogen KNO_3_ as the nitrogen source. In the medium where KNO_3_ was substituted with the preferred nitrogen NH_4_Cl, only a small accumulation of pri-SMTP was observed (Figure 6B,D). No pri-SMTP was produced in the medium where sucrose was replaced by *myo*-inositol (Figure 6B,D). Notably, virtually no peaks were found in the chromatograms for the analysis of the extracts from cells cultured on NH_4_Cl or *myo*-inositol (Figure 6D), suggesting an entire arrest of the SMTP biosynthetic pathway under these nutritional conditions. Unlike the drastic changes in pri-SMTP accumulation, significant levels of pri-SMTP oxidase activity were observed among the different nutritional conditions (Figure 6C).

**Figure 7:**
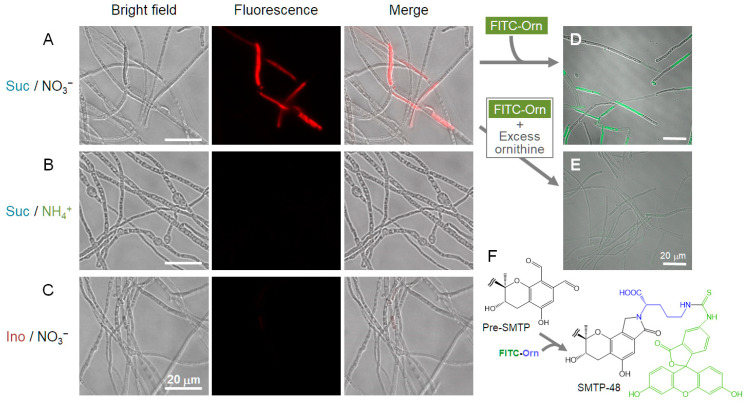
Localization of pri-SMTP biosynthesis. Fluorescence microscopic localization of pri-SMTP accumulation in *S. microspora* grown on different nutrients: Suc/KNO_3_ (**A**), Suc/NH_4_^+^ (**B**), and Ino/KNO_3_ (**C**) (excitation at 560 nm and emission at 630 nm). Medium abbreviations are the same as those in Figure 6. Pri-SMTP fluorescence is observed in particular, but not in all, septate cells grown on Suc/KNO_3_ (**A**). Essentially no fluorescent cells are visible in the cells grown on Suc/NH_4_^+^ (**B**) or Ino/KNO_3_ (**C**) medium, consistent with the UPLC data (see Figure 6D). Fluorescence microscopic localization of pre-SMTP formation (**D**) and its conversion to SMTP-48 (**E**) (excitation at 470 nm and emission at 525 nm). (**F**) The formation of a fluorescent SMTP analog, SMTP-48, from pre-SMTP via the conjugation with FITC-Orn. When FITC−Orn was added to the Suc/KNO_3_ culture on Day 4, particular septate cells that were positive in fluorescence were detected (**D**), demonstrating a specified localization of pre-SMTP generation and its conversion to SMTP, which appears similar to the localization of pri-SMTP shown in panel A. The addition of an excess of L-ornithine, along with FITC−Orn, decreases the fluorescence labeling of cells (**E**).

Pri-SMTP localization was visualized using fluorescence microscopy (Figure S6A,B). Pri-SMTP accumulates in particular septate cells (Figure 7A and Figure S6B). Pri-SMTP accumulation disappeared after the addition of L-ornithine, which led to SMTP-7 formation, verifying the pri-SMTP fluorescence observation (Figure S6C). None of the cells grown on NH_4_Cl or *myo*-inositol exhibited fluorescence (Figure 7B,C). The addition of FITC−Orn, which afforded SMTP-48 upon conjugation with pre-SMTP, labelled particular septate cells (Figure 7D) just like those accumulated pri-SMTP (Figure 7A). The FITC−Orn-mediated SMTP-48 formation was quenched by excess L-ornithine, demonstrating the specificity of the fluorescence observation (Figure 7E). Thus, the pri-SMTP-accumulation site can provide a place for pri-SMTP oxidation (i.e., pre-SMTP generation) and amine-capturing by the resulting pre-SMTP (i.e., SMTP formation).

**Figure 8:**
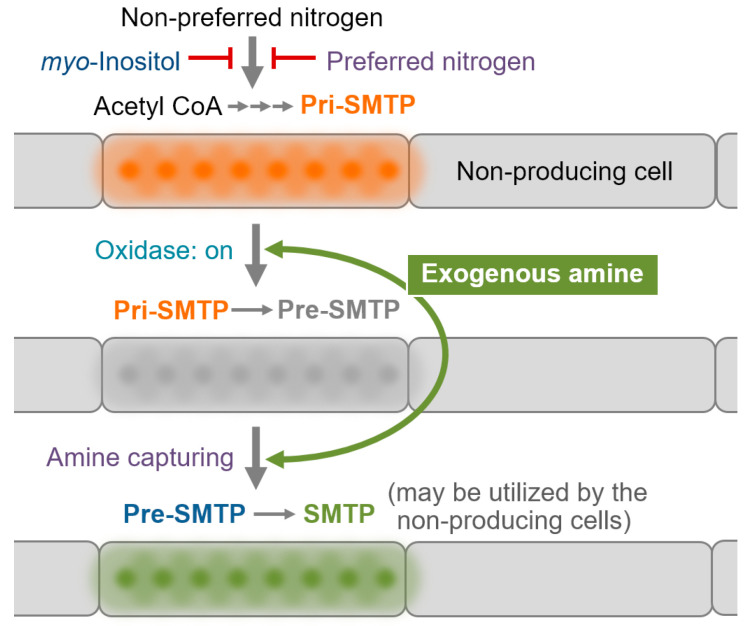
Possible mechanism fo the regulation of SMTP biosynthesis and its nutritional role. See text for details of the explanation of the scheme. Please note that the exogenous amine fed to the culture did not induce pri-SMTP oxidase (Figure 4C), and the amine added to the cell-free enzyme assay did not activate the oxidase activity (Figure 4E,F). However, pri-SMTP was consumed when the exogenous amine was added to the culture, but not when no amine was added (Figure 2E,F). Therefore, we assume that exogenous amines can trigger the oxidase reaction via a mechanism other than induction or direct activation, whereas the mechanism that triggers oxidase reaction is still elusive.

Taken together, these data suggest that SMTP biosynthesis is regulated at least by the following three distinct mechanisms: (i) nitrogen- and carbon nutrients-dependent regulation of the pathway to pri-SMTP; (ii) localization of the biosynthesis/accumulation of pri-SMTP; and (iii) amine-regulated oxidation of pri-SMTP to pre-SMTP (Figure 8). Generally, enzymes for secondary metabolism are compartmentalized at conserved subcellular sites or organelles [22,23]. The localization of the pri-SMTP storage in particular septate mycelia in *S. microspora* appears to be unique, and this type of compartmentalization can provide a mechanism to quarantine the highly reactive pre-SMTP within specified cells where amine-capturing by pre-SMTP should predominantly occur. Furthermore, the pri-SMTP localization may play a role in regulated pri-SMTP oxidation in response to exogenous amines, whereas further studies are needed to clarify a detailed mechanism.

### 3.7. Nitrogen-Related Nutritional Aspects of the Biosynthesis of SMTP

Metabolic regulations by nutrients, particularly nitrogen and carbon catabolite repression in bacteria and fungi, is a strategy for accommodating changes in the nutrient sources [24]. Ammonium and glucose are preferred nutrients in filamentous fungi [25]. Generally, nitrogen metabolite repression ensures the preferential utilization of ammonium or L-glutamine over alternative nitrogen sources, and carbon catabolite repression acts to repress genes for the assimilation of alternative carbon sources. *S. microspora* efficiently produces SMTPs on the preferred carbon sources glucose and sucrose. It appears that *myo*-inositol is an alternative carbon source in *S. microspora* based on the poor biomass yield compared to that in the sucrose culture (Figure 6A). Thus, the suppression of SMTP biosynthesis in the *myo*-inositol culture is unlikely to represent the canonical carbon catabolite repression [26]. Since *myo*-inositol is an essential nutrient serving as a precursor to phosphatidylinositol and its derivatives in eukaryotes, including fungi [27], the *myo*-inositol suppression of pri-SMTP synthesis may have nutritional implications.

The ammonium suppression of pri-SMTP synthesis (Figure 6B) may be involved in broad metabolic changes due to nitrogen catabolite repression. SMTP is produced under conditions where the amounts of preferred nitrogen sources are limited. Generally, *Stachybotrys* inhabits low-nitrogen environments [28]. It is notable that the amine-regulated pri-SMTP oxidation/pre-SMTP generation is coupled with subsequent SMTP formation (i.e., amine-capturing with pre-SMTP) (Figure 8). This mechanism can play a role in the nitrogen acquisition/assimilation strategy in *S. microspora*.

## 4. Conclusions

The discovery of the key intermediate, pri-SMTP, and the enzyme that oxidizes pri-SMTP to amine-reactive pre-SMTP, leads to an understanding of the biological significance of the production in *S. microspora* of the secondary metabolites SMTPs, which exhibit significant pharmacological activities. Pri-SMTP formation is under the control of nitrogen and carbon nutrients, and the limitation of preferred nitrogen induces its biosynthesis. Pri-SMTP accumulates in particular septate mycelia, and this localization limits the production of the highly reactive pre-SMTP in specified cells. The oxidation of pri-SMTP occurs when exogenous amine is available to the cells. These mechanisms enable *S. microspora* to acquire and store alternative nitrogen sources in the case of preferred nitrogen deficiency (Figure 8). An analysis of the whole genomic DNA sequences of *S. microspora* is underway to delineate the details of the regulation of SMTP biosynthesis in conjunction with its nutritional aspects.

## Data Availability

The data presented in this study are available in this article and in the Supplementary Material.

## Supplementary Materials

The following are available online at https://www.mdpi.com/article/10.3390/jof8090975/s1. Figure S1: ^1^H- and ^13^C-NMR spectra of pri-SMTP; Figure S2: Two-dimensional NMR spectroscopic analysis of pri-SMTP; Figure S3: Peptides mapped via the peptide mass fingerprint analysis of the 51 kDa protein; Figure S4: Localization of pri-SMTP oxidase-like gene in scaffold1638 of *S. chlorohalonata* IBT 40285 (GenBank KL660690.1), and putative functions of the genes therein; Figure S5: Identification of a cluster of genes possibly involved in the biosynthesis of ilicicolin B/SMTP-like metabolites in scaffold432 in the *S. chlorohalonata* IBT 40285 genome; Figure S6: Fluorescence microscopic visualization of pri-SMTP-accumulating cells; Table S1: Assignment of the ^1^H- and ^13^C-NMR spectral data for pri-SMTP; Table S2: Physicochemical properties of pri-SMTP; Table S3: Summary of the purification of pri-SMTP oxidase; Table S4: Parameters for the peptide mass fragment analysis; Table S5: Supporting peptides.
