# Supplementary material for: Amine-Regulated pri-SMTP Oxidation in SMTP Biosynthesis in Stachybotrys: Possible Implication in Nitrogen Acquisition
Source: J Fungi (Basel). 2022 Sep 18;8(9):975. doi: 10.3390/jof8090975 (PMC9502257; doi:10.3390/jof8090975)
Supplement: Supplementary file 1 [file jof-08-00975-s001.zip › jof-1913340-supplementary.pdf]

Supplementary Materials

Amine-Regulated pri-SMTP Oxidation in SMTP Biosynthesis in *Stachybotrys*: Possible Implication in Nitrogen Acquisition

Ryota Iwama [1], Yu Sasano [1], Taichi Hiramatsu [1], Shinya Otake [1], Eriko Suzuki [1], and Keiji Hasumi [1,2,*]

[1] Department of Applied Biological Science, Tokyo University of Agriculture and Technology, Fuchu, Japan
[2] Department of Research and Development, TMS Co., Fuchu, Japan
* Correspondence: hasumi@cc.tuat.ac.jp

Contents

Figure S1: ^{1}H- and ^{13}C-NMR spectra of pri-SMTP

Figure S2: Two-dimensional NMR spectroscopic analysis of pri-SMTP

Figure S3: Peptides mapped via the peptide mass fingerprint analysis of the 51 kDa protein

Figure S4: Localization of pri-SMTP oxidase-like gene in scaffold1638 of *S. chlorohalonata* IBT 40285 (GenBank KL660690.1), and putative functions of the genes therein

Figure S5: Identification of a cluster of genes possibly involved in the biosynthesis of ilicicolin B/SMTP-like metabolites in scaffold432 in the *S. chlorohalonata* IBT 40285 genome

Figure S6: Fluorescence microscopic visualization of pri-SMTP-accumulating cells

Table S1: Assignment of the ^{1}H- and ^{13}C-NMR spectral data for pri-SMTP

Table S2: Physicochemical properties of pri-SMTP

Table S3: Summary of the purification of pri-SMTP oxidase

Table S4: Details of the search setting and supporting peptide results

Table S5: Supporting peptides

Figure S1

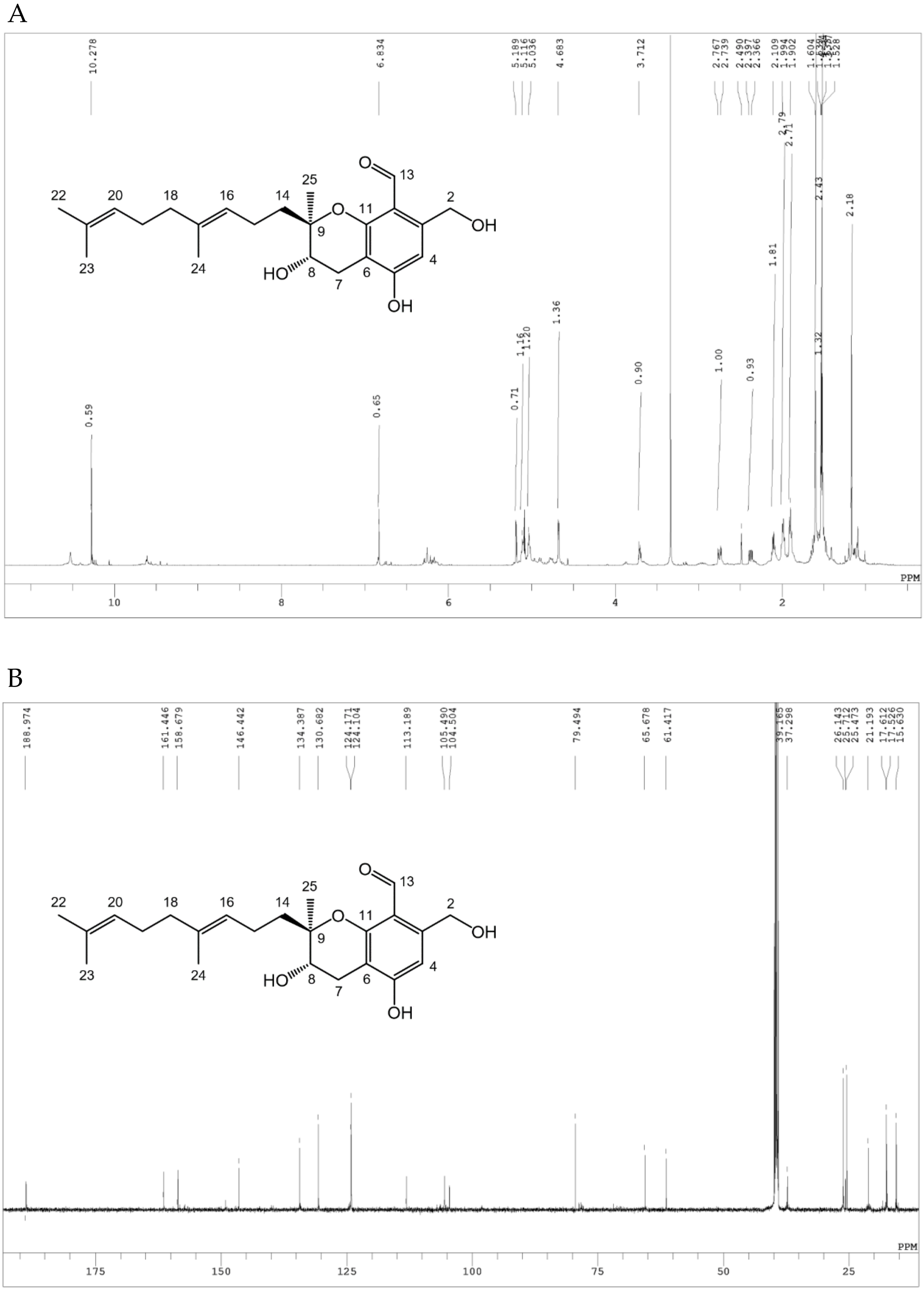

Figure S1. ¹H- and ¹³C-NMR spectra of pri-SMTP. The ¹H-NMR (A) and ¹³C-NMR (B) spectra were obtained using dimethyl sulfoxide-*d*$_6$ (δ_H 2.50 ppm; δ_C 39.52 ppm).

Figure S2

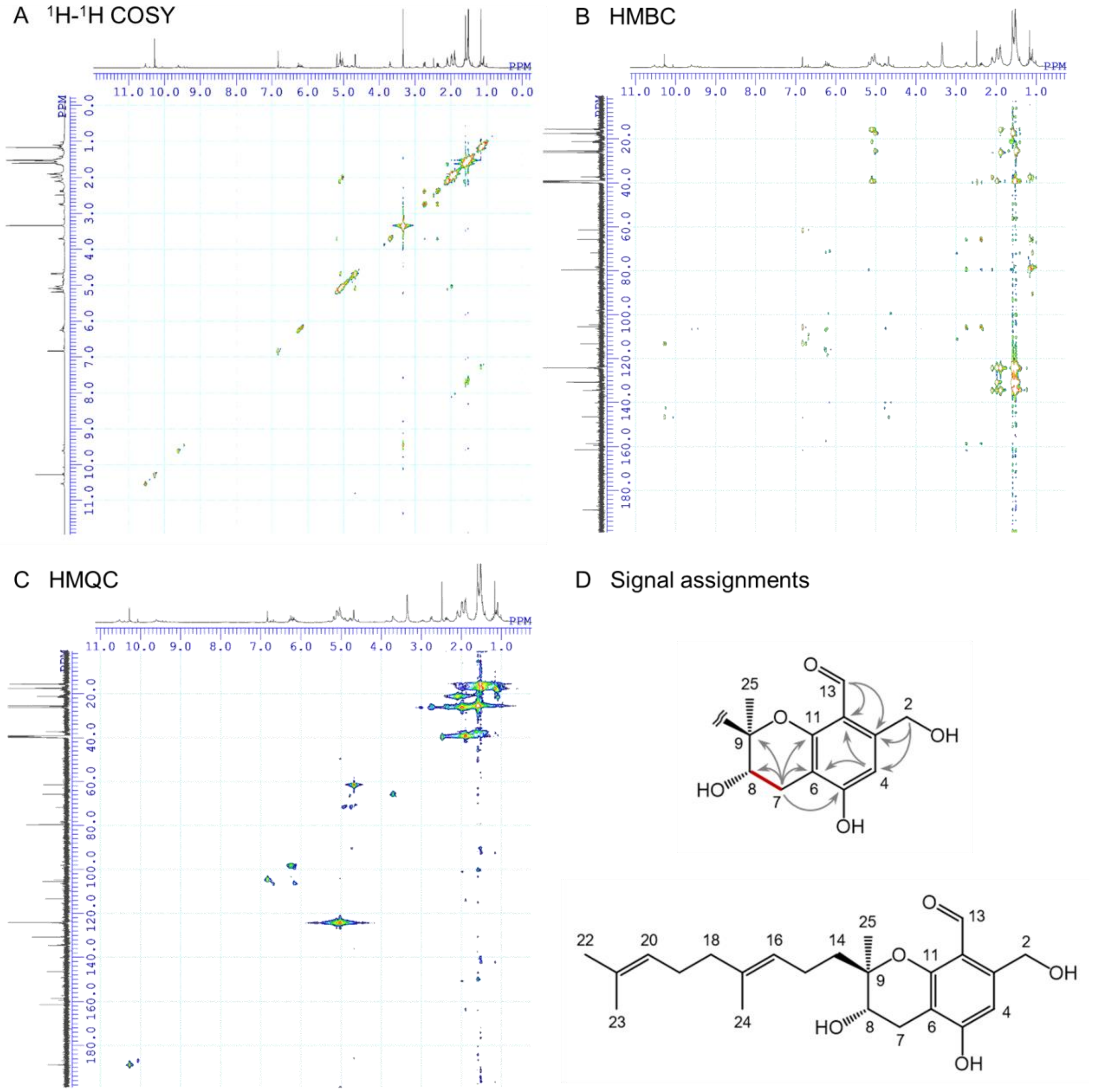

Figure S2. Two-dimensional NMR spectroscopic analysis of pri-SMTP. (A) ^{1}H-^{1}H correlation spectroscopy (COSY), (B) ^{1}H-detected multi-bond heteronuclear multiple quantum coherence (HMBC), and (C) ^{1}H-detected multiple quantum coherence (HMQC) spectra of pri-SMTP in dimethyl sulfoxide-d_6. (D) Assignment of the structure based on the combination of the coupling data. ***Arrows***, HMBC long-range couplings; ***red bond***, COSY vicinal coupling.

Figure S3

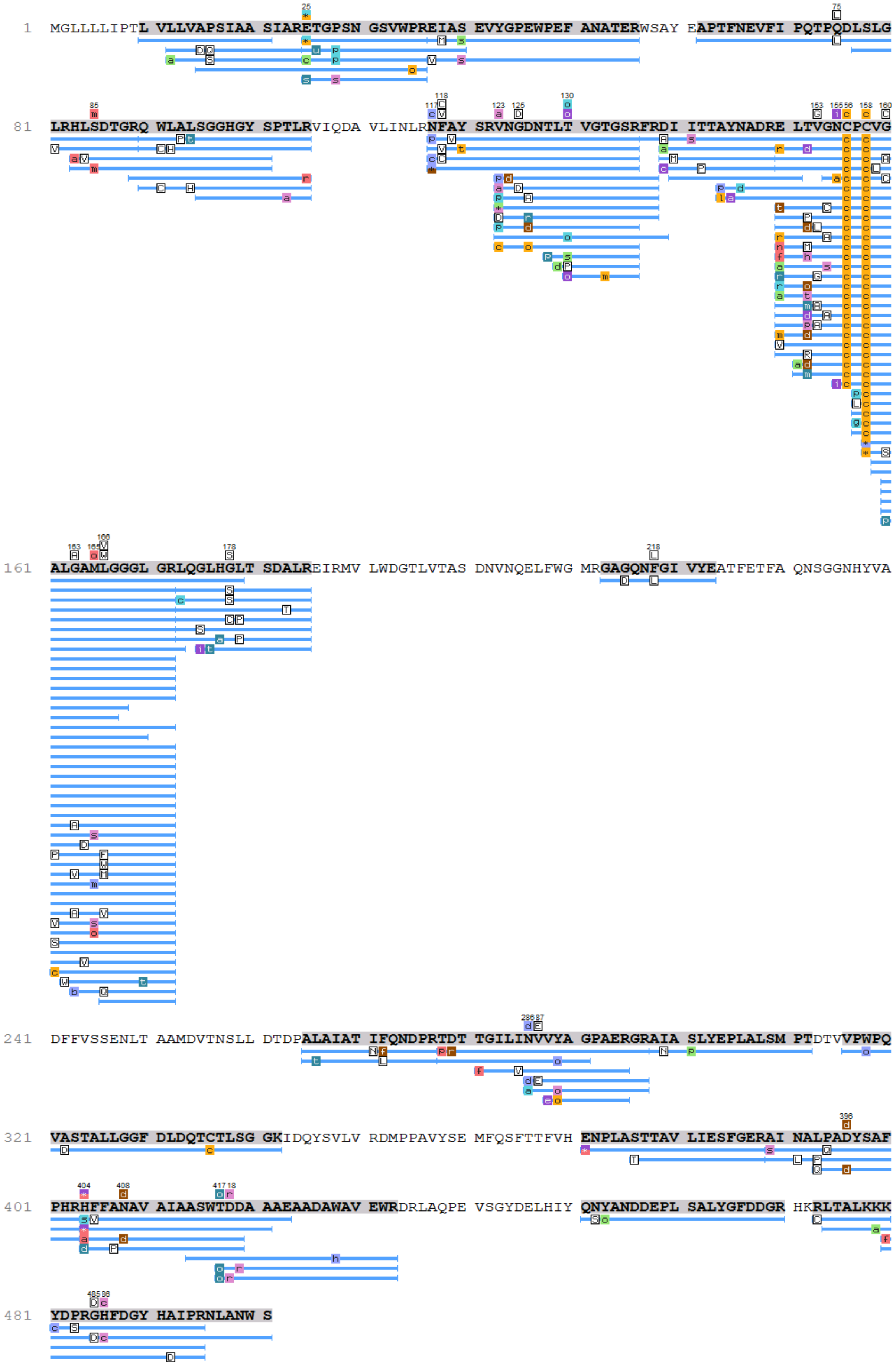

To be continued

Figure S3 (continued)

- d 5-dimethylaminonaphthalene-1-sulfonyl (+233.05)
- d Deoxy (-15.99)
- d Diethylation (+56.06)
- d 2,4-diacetamido-2,4,6-trideoxyglucopyranose (+228.11)
- d Deamidation followed by esterification with ethanol (+29.04)
- d Dehydration (-18.01)
- d Dimethylation (+28.03)
- e Ethylation (+28.03)
- f Fluorination (+17.99)
- f Formylation (+27.99)
- g Glycosyl-L-hydroxyproline (+148.04)
- h Hexose (+162.05)
- h HexNAcylation (ST) (+203.08)
- i ISD (z+2)-series (-15.01)
- l Levuglandinyl-lysine anhyropyrrole adduct (+298.19)
- m Methylation (+14.02)
- m Methylphosphonylation (+77.99)
- m Malonylation (+86.00)
- m Methionine replacement by azido homoalanine (-4.99)
- n Naphthalene-2,3-dicarboxaldehyde (+175.04)
- o Oxidation or Hydroxylation (+15.99)
- o Oxidation to nitro (+44.99)
- o O-Diisopropylphosphorylation (+164.06)
- o O-Isopropylphosphorylation (+122.01)
- o Octanoyl (+126.10)
- o O-Diethylphosphorylation (+136.03)
- o O-Ethylphosphorylation (+108.00)
- o O-Pinacolylmethylphosphonylation (+162.08)
- o Oxidation (M) (+15.99)
- p Phosphorylation (STY) (+79.97)
- p Propionamide (K, X@N-term) (+71.04)
- p Pyridylacetyl (+119.04)
- p Pyrrolidone from Proline (-27.99)
- p Piperidination (+68.06)
- p Pyrophosphorylation (ST) (+159.93)
- p Phospho-propargylamine (+117.00)
- r Replacement of proton by potassium (+37.96)
- r Replacement of proton by lithium (+6.01)
- r Replacement of 2 protons by nickel (+55.92)
- r Replacement of proton by silver (+105.90)
- r Replacement of 2 protons by magnesium (+21.97)
- r Replacement of proton with ammonium ion (+17.03)
- s Sulfation (+79.96)
- s 3-sulfanylpropanoyl (+88.00)
- s 4-sulfophenyl isothiocyanate (+214.97)
- s 3-Sulfobenzoic succinimidyl ester (+183.98)
- t Tyrosine oxidation to 2-aminotyrosine (+15.01)
- t Thiophosphorylation (+95.94)
- t Trifluoroleucine (+53.97)
- t Tri nitro benzene (+210.99)
- u Ubiquitin (+114.04)
- * Amidination of lysines or N-terminal amines with methyl acetimidate (+41.03), 3-sulfanylpropanoyl (+88.00)
- * Carboxymethyl (+58.01), Hexose (+162.05)
- * Carboxymethyl (+58.01), N-Succinimidyl-2-morpholine acetate (+127.06)
- * Ethylation (+28.03), Aminoethylbenzenesulfonylation (+183.04)
- * ISD (z+2)-series (-15.01), Sodium adduct (+21.98)
- * 3-methyl-2-pyridyl isocyanate (+134.05), Methylation (+14.02)
- * Piperidination (+68.06), Dimethylation (+28.03)
- D A->D
- P A->P
- S A->S
- T A->T
- V A->V
- A D->A
- V E->V
- C F->C
- L F->L
- V F->V
- A G->A
- C G->C
- D G->D
- S G->S
- V G->V
- M I->M
- N I->N
- V I->V
- F L->F
- H L->H
- M L->M
- P L->P
- Q L->Q
- V L->V
- W L->W
- L N->L
- S N->S

Figure S3. Peptides mapped via the peptide mass fingerprint analysis of the 51 kDa protein. The peptide mass fingerprint analysis was performed using PEAKS DB (Bioinformatics Solutions Inc., Waterloo, ON, Canada). Annotation symbols are given in the lower panel. Details of the search setting and supporting peptide results are shown in Table S4 and Table S5, respectively.

Figure S4

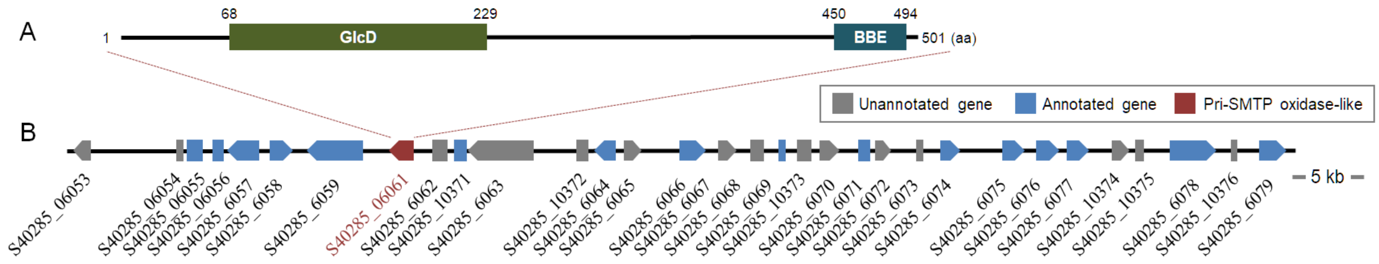

Locus Tag	Putative Function
S40285_06055	Acetyl esterase/lipase
S40285_06056	SMP-30/Gluconolaconase/LRE-like region
S40285_06057	C2H2 Zn finger
S40285_06058	Nucleobase-cation-symport-1 transporter
S40285_06059	choice-of-anchor B domain
S40285_06061	FAD/FMN-containing dehydrogenase (**pri-SMTP oxidase-like**)
S40285_10371	Ribonuclease H-like superfamily
S40285_06064	WD40 domain
S40285_06066	Cytochrome P450
S40285_06069	Cupredoxin superfamily
S40285_06071	Short chain dehydrogenase
S40285_06074	Glycosyl hydrolase
S40285_06075	GAL4-like Zn2Cys6 binuclear cluster DNA-binding domain
S40285_06076	Cytochrome P450
S40285_06077	The Major Facilitator Superfamily
S40285_06078	Nucleotidyltransferase domain of DNA polymerase beta and similar proteins
S40285_06079	β-Lactamase

Figure S4. Localization of pri-SMTP oxidase-like gene in scaffold1638 of *S. chlorohalonata* IBT 40285 (GenBank KL660690.1), and putative functions of the genes therein. (A) Functional domains predicted for pri-SMTP oxidase. The prediction was based on the sequence of an orthologue of pri-SMTP oxidase gene identified in scaffold1638 (GenBank KL660690.1) of *S. chlorohalonata* IBT 40285 genomic DNA sequences [21]. GlcD, glycolate oxidase subunit D superfamily domain; BBE, berberine and berberine-like superfamily domain found in berberine bridge and berberine bridge-like enzymes in a class of FAD-linked oxidases. (B and the *lower table*) Scaffold1638 contains >30 possible genes that seems to be unrelated to secondary metabolism. The annotation was based on the functional domain searches [19] using the predicted amino acid sequences of the genes in scaffold1638 (GenBank KL660690.1). A search for secondary metabolite biosynthesis genes using antiSMASH fungal version (ver. 6.0.1) [20] failed to find any candidates in this scaffold.

Figure S5

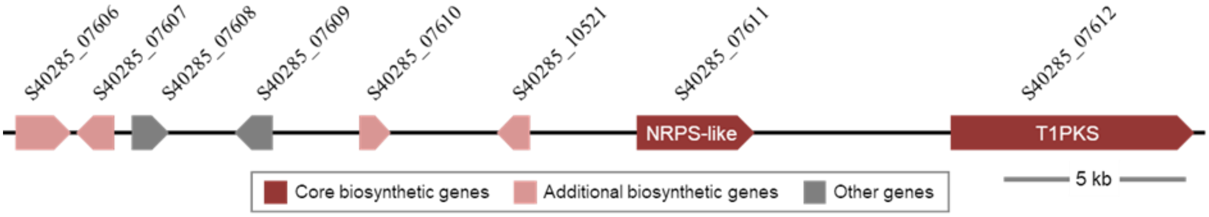

Locus Tag	Putative Function
S40285_07606	Esterase, PHB depolymerase family
S40285_07607	Oxidoreduntase, classical (c) SDR, subgroup 1
S40285_07608	Isomerase; may be involved in myo-inositol catabolism
S40285_07609	Copper-dependent lytic polysaccharide monooxygenases
S40285_07610	Oxidoreduntase, classical (c) SDR
S40285_10521	Prenyltransferase
S40285_07611	Non-ribosomal peptide synthase-like
S40285_07612	Polyketide synthase

Figure S5. Identification of a cluster of genes possibly involved in the biosynthesis of ilicicolin B/SMTP-like metabolites in scaffold432 in the *S. chlorohalonata* IBT 40285 genome. A putative SMTP biosynthesis gene cluster is found in a different scaffold than the scaffold that contains pri-SMTP oxidase-like gene (Figure S4). The search for a cluster of secondary metabolite biosynthesis genes was performed using antiSMASH fungal version (ver. 6.0.1) [20]. The annotation was based on the functional domain searches [19] using the predicted amino acid sequences of the genes in scaffold432 (GenBank KL659535.1). NRPS, non-ribosomal peptide synthase; T1PKS, type I polyketide synthase.

Figure S6

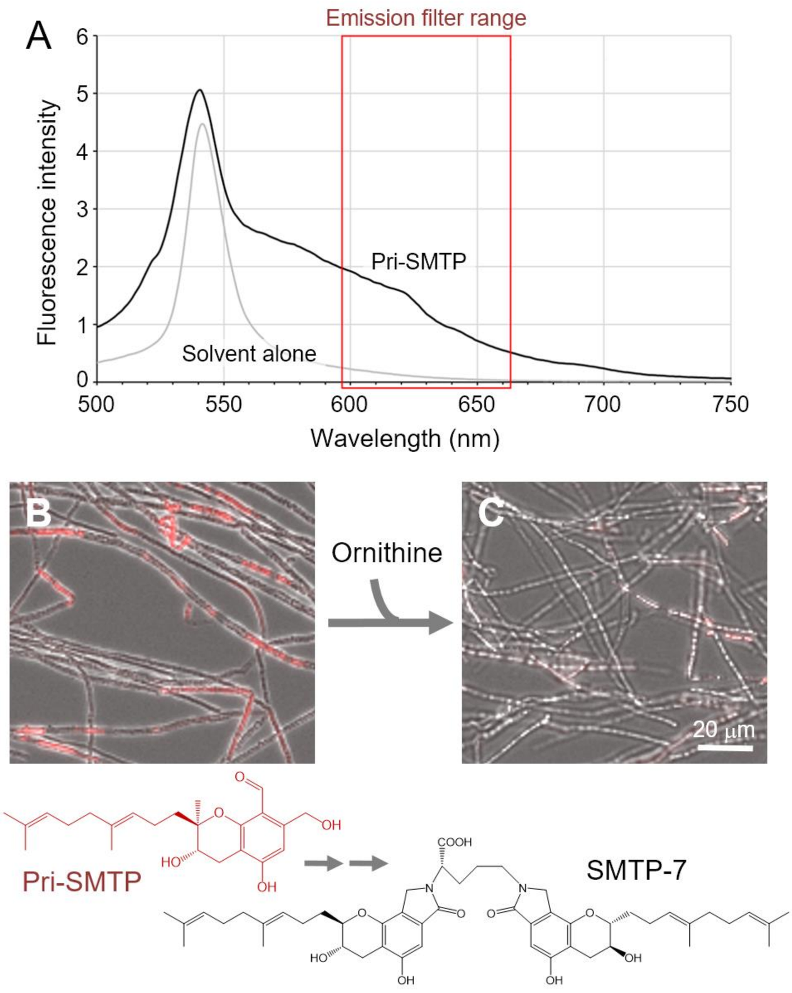

Figure S6. Fluorescence microscopic visualization of pri-SMTP-accumulating cells. (A) Fluorescence spectrum of pri-SMTP in in MeCN–0.1% (vol vol^{-1}) formic acid aq. (7:1) with an excitation at 540 nm. The range of the emission wavelength acquirable using a BZ-X TxasRed filter, with median excitation and emission wavelengths at 560 and 630 nm, respectively, is shown. (B) Fluorescence microscopic analysis of the localization of pri-SMTP in *S. microspora*. Particular septate cells are positive in fluorescence when using a BZ-X TexasRed filter. (C) Fluorescence largely disappears after L-ornithine feeding, in consistent with the decrease in the pri-SMTP levels as determined via UPLC analysis (see Figure 2). The structures of pri-SMTP and SMTP-7 are shown.

Table S1. Assignment of the ^{1}H- and ^{13}C-NMR spectral data for pri-SMTP.

No.	δ_C	δ_H	
1		5.19	(OH, 1 H, d, J = 4.8)
2	61.4	4.68	(2 H, d, J = 4.8)
3	146.4		
4	104.1	6.83	(1 H, s)
5	161.4		
6	105.5		
7	26.1	2.75	(1 H, dd, J = 6.0, 16.2)
		2.38	(1 H, dd, J = 8.4, 17.4)
8	65.7	3.71	(1 H, m)
9	79.5		
11	158.7		
12	113.2		
13	189.0	10.28	(1 H, s)
14	37.3	1.54	(2 H, m)
15	21.2	2.11	(2 H, m)
16	124.2	5.09	(1 H, m)
17	134.4		
18	39.2	1.90	(2 H, m)
19	25.7	1.99	(2 H, m)
20	124.1	5.01	(1 H, m)
21	130.7		
22	25.5	1.60	(3 H, s)
23	17.5	1.52	(3 H, s)
24	15.6	1.53	(3 H, s)
25	17.6	1.12	(3 H, s)

Table S2. Physicochemical properties of pri-SMTP.

Appearance	Brown oil or white powder
Molecular formula	$C_{23}H_{32}O_5$
ESI-MS (m/z)	
Found	387.2177 (M – H)$^-$
Calcd	387.2177 for $C_{23}H_{31}O_5$
UV λ_{max} nm (ε)	
MeOH−0.1% formic acid, aq. 21:4 (v/v)	208 (43,900), 228 (9,880, sh), 271 (1,760), 280 (1,780)
MeCN−0.1% formic acid, aq. 7:1 (v/v)	230 (11,600, sh), 285 (7,440), 320 (3,900, sh)
IR ν_{max} (KBr) cm^{-1}	3348, 2967, 2919, 1631, 1606, 1593, 1503, 1457, 1401, 1377, 1343, 1296, 1238, 1160, 1100, 1007

Table S3. Summary of the purification of pri-SMTP oxidase.

Step	Total protein (mg)	Total activity (nmol min^{-1})	Specific activity (nmol mg^{-1} min^{-1})	Yield (%)
Cytosol	340	6,107	18.0	100
Ammonium sulfate	64.9	4,637	71.4	75.9
1st Hiprep Q	10.9	737	67.6	12.1
2nd Hiprep Q	2.75	686	249	11.2

Pri-SMTP oxidase activity was obtained by measuring the consumption of pri-SMTP.

Table S4. Parameters for the peptide mass fragment analysis.

**Instrument parameters**	
Fractions: sample2.raw	
Ion Source: ESI(nano-spray)	
Fragmentation Mode: CID, CAD (y and b ions)	
MS Scan Mode: FT-ICR/Orbitrap	
MS/MS Scan Mode: Linear Ion Trap	
**Search parameters**	
Search Engine Name: PEAKS	Max Variable PTM Per Peptide: 3
Parent Mass Error Tolerance: 10.0 ppm	Database: NCBI_S_chlorohalonata
Fragment Mass Error Tolerance: 0.5 Da	Taxon: All
Precursor Mass Search Type: monoisotopic	Searched Entry: 10720
Enzyme: Trypsin	FDR Estimation: Enabled
Max Missed Cleavages: 3	De novo score (ALC%) threshold: 15
Digest Mode: Semispecific	Peptide hit threshold (-10logP): 30.0
Fixed Modifications:	Peaks run ID: 14
Carboxymethyl: 58.01	Merge Options: no merge
Variable Modifications:	Precursor Options: corrected
Oxidation (M): 15.99	Charge Options: no correction
Deamidation (NQ): 0.98	Filter Quality: >0.65
Acetylation (K): 42.01	Filter Charge: 1−6
Acetylation (Protein N-term): 42.01	Process: true
Acetylation (N-term): 42.01	Associate chimera: yes
Amidation: −0.98	
Beta-methylthiolation: 45.99	
Biotinylation: 226.08 and 476 more	

Table S5. Supporting peptides.

Entry	Supporting peptide	Uniq	−10 lgP	Mass	Length	ppm	m/z	z	RT	Scan	Area	Start	End	PTM
1	C.VGALGAMLGGGLGR.L	Y	78.09	1227.6758	14	0.3	614.8453	2	20.67	3811	2.75E+06	159	172	
2	V.GALGAMLGGGLGR.L	Y	74.94	1128.6073	13	-0.5	565.3107	2	19.52	3405	2.95E+06	160	172	
3	R.ELT(-18.01)VA(sub G)NC(+58.01)PC(+58.01)VGALGAMLGGGLGR.L	Y	73.83	2256.0701	23	6.8	1129.0499	2	24.73	5188	4.61E+06	150	172	Dehydration; Carboxymethyl
4	E.L(+41.03)T(-15.99)VGNC(+58.01)PC(+58.01)VGALGAMLGGGLGR.L	Y	72.38	2156.054	22	9.9	1079.0449	2	25.44	5375	4.06E+06	151	172	Amidination of lysines or N-terminal amines with methyl acetimidate; Deoxy; Carboxymethyl
5	R.V(+27.99)(sub E)LTVGNC(+58.01)PC(+58.01)VGALGAMLGGGLGR.L	Y	71.33	2258.0857	23	6.5	1130.0575	2	25.29	5480	3.38E+06	150	172	Formylation; Carboxymethyl
6	R.ELT(-15.99)L(sub V)GNC(+58.01)PC(+58.01)VGALGAMLGGGLGR.L	Y	68.68	2258.0857	23	6.5	1130.0575	2	25.29	5422	1.20E+07	150	172	Deoxy; Carboxymethyl
7	R.E(+175.04)LM(sub T)VGNC(+58.01)PC(+58.01)VGALGAMLGGGLGR.L	Y	59.23	2465.1001	23	0.4	822.7076	3	25.1	5540	1.36E+06	150	172	Naphthalene-2,3-dicarboxaldehyde; Carboxymethyl
8	H.FDGYHP(sub A)IPR.N	Y	53.56	1100.5403	9	-0.8	551.277	2	15.14	1818	1.97E+05	487	495	
9	R.E(+14.02)LT(-15.99)VGNC(+58.01)PC(+58.01)VGALGAMLGGGLGR.L	Y	52.92	2258.0857	23	6	753.707	3	25.29	5319	8.64E+06	150	172	Methylation; Deoxy; Carboxymethyl
10	R.ELTVGNC(+58.01)PC(+58.01)L(sub V)GALGAMLGGGLGR.L	Y	51.85	2274.0806	23	6.7	1138.0552	2	21.33	3963	9.50E+05	150	172	Carboxymethyl
11	R.ELT(+79.97)A(sub V)GNC(+58.01)PC(+58.01)VGALGAMLGGGLGR.L	Y	48.06	2312	23	7.3	771.6796	3	25.29	5322	3.36E+06	150	172	Phosphorylation (STY); Carboxymethyl
12	R.L(+57.02)QGLHS(sub G)LTSDALR.E	Y	46.75	1466.7841	13	0.1	734.3994	2	17.51	2636	6.06E+06	173	185	Carbamidomethylation (DHKE, X@N-term)
13	R.ELR(sub T)VGNC(+58.01)PC(+58.01)VGALGAMLGGGLGR.L	Y	46.01	2315.1184	23	-0.2	772.7133	3	25.32	5322	3.17E+05	150	172	Carboxymethyl
14	W.T(+162.08)DD(+17.03)AAAEAADAWAVEWR.D	Y	44.95	2025.9197	17	1.9	1013.969	2	28.16	6255	7.26E+05	417	433	O-Pinacolylmethylphosphonylation; Replacement of proton with ammonium ion
15	R.LQGLHGLTSDT(sub A)LR.E	Y	43.19	1409.7627	13	0.9	705.8893	2	20.68	3790	4.15E+08	173	185	
16	R.LQGLHC(sub G)P(sub L)TSDALR.E	Y	41.48	1409.7085	13	-2.7	705.8596	2	21.13	3916	2.08E+04	173	185	
17	V.GALGAM(+15.99)LGGGLGR.L	Y	40.77	1144.6023	13	-0.1	573.3083	2	16.57	2288	9.93E+05	160	172	Oxidation (M)
18	W.T(+162.08)D(+17.03)DAAAEAADAWAVEWR.D	Y	40	2025.9197	17	1.9	1013.969	2	28.16	6213	7.26E+05	417	433	O-Pinacolylmethylphosphonylation; Replacement of proton with ammonium ion
19	K.KYDPRGHFDGYHD(sub A)IPR.N	Y	38.98	1971.934	16	2.5	986.9767	2	25.89	5548	3.01E+06	480	495	
20	R.ELTVGNC(+58.01)PC(+58.01)VA(sub G)ALGAMLGGGLGR.L	Y	38.21	2274.0806	23	6.4	759.039	3	21.33	4068	2.48E+06	150	172	Carboxymethyl
21	R.LQGLHS(sub G)LTSDALR.E	Y	36.46	1409.7627	13	1.4	705.8896	2	21.03	3916	2.86E+08	173	185	
22	R.E(+21.97)LT(-18.01)VGNC(+58.01)PC(+58.01)VGALGAMLGGGLGR.L	Y	36.14	2264.0239	23	-1.5	567.0124	4	20.82	3807	9.00E+04	150	172	Replacement of 2 protons by magnesium; Dehydration; Carboxymethyl
23	R.LQS(sub G)LHGLTSDALR.E	Y	33.94	1409.7627	13	-0.4	470.928	3	39.28	8454	0	173	185	
24	M.LGGGLGR.L	N	33.72	628.3656	7	0.2	315.1901	2	12.64	1058	7.47E+05	166	172	
25	R.QC(sub W)H(sub L)ALSGGHGYSPTLR.V	Y	32.8	1682.7947	16	4	842.408	2	38.7	8370	0	90	105	
26	E.APTFNEVFIPQTPL(sub Q)DLSLGLRHLSDTGR.Q	Y	32.69	3093.6248	28	-1.1	1032.2144	3	27.48	6128	3.86E+05	62	89	
27	L.G(+226.08)AMQ(sub L)GGGLGR.L	Y	32.27	1128.5168	10	5.6	565.2689	2	20.13	3581	1.02E+06	163	172	Biotinylation
28	G.A(+58.01)LGAMLGGGLGR.L	Y	30.42	1129.5913	12	-3.2	565.8011	2	20.16	3581	7.11E+04	161	172	Carboxymethyl (KW, X@N-term)
29	V.APSIAASIARETGPSNGSVWP(+15.99)R.E	Y	27.91	2239.1345	22	-0.6	747.385	3	30.25	6806	9.78E+05	15	36	Oxidation or Hydroxylation
30	R.N(+68.06)FV(sub A)YSRVNGDNTLTVGTGSR.F	Y	25.92	2224.1235	20	-3.4	742.3793	3	20.13	3646	1.29E+07	117	136	Piperidination

(to be continued)

(continued)

31	R.ELP(sub T)VGNC(+58.01)PC(+58.01)VGALGAMLGGGLGR.L	Y	25.53	2256.0701	23	6.8	1129.0499	2	24.73	5137	6.96E+05	150	172	Carboxymethyl
32	R.ELT(+77.99)A(sub V)GNC(+58.01)PC(+58.01)VGALGAMLGGGLGR.L	Y	25.33	2310.0208	23	-8.6	578.5075	4	24.73	5178	3.28E+05	150	172	Methylphosphonylation; Carboxymethyl
33	C.VGALA(sub G)AMV(sub L)GGGLGR.L	Y	25.14	1227.6758	14	0.3	614.8453	2	20.67	3866	2.75E+06	159	172	
34	V.GN(-17.03)C(+58.01)PC(+58.01)VC(sub G)ALGAMLGGGLGR.L	Y	24.14	1846.7834	19	-2.9	616.6	3	26.36	5828	7.85E+05	154	172	Ammonia-loss (N); Carboxymethyl
35	R.T(+117.00)D(+6.01)TTGILINVVYAGPAERGR.A	Y	24.08	2225.1182	20	-1.3	742.7124	3	21.56	4251	8.04E+05	278	297	Phosphopropargylamine; Replacement of proton by lithium
36	R.D(+43.99)IIP(sub T)TAYNADR.E	Y	23.37	1291.6044	11	-8.1	646.8042	2	23.6	4947	3.33E+05	139	149	Carboxylation (DKW)
37	R.LQGLH(+28.03)GP(sub L)TSDALR.E	Y	23.29	1391.7521	13	0.8	464.925	3	17.55	2645	0	173	185	Acetaldehyde +28
38	R.LTALKK(+41.03)KYDPR.G	Y	23	1372.819	11	-2.5	458.6125	3	22	4257	1.18E+04	474	484	Amidination of lysines or N-terminal amines with methyl acetimidate
39	T.T(+27.99)GILV(sub I)NVVYAGPAER.G	Y	22.99	1585.8463	15	1	529.6232	3	25.01	5259	9.21E+04	281	295	Formylation
40	C.P(-27.99)C(+58.01)VGALGAM(+88.00)LGGGLGR.L	Y	22.85	1545.7466	16	7.2	516.2598	3	27.99	6188	0	157	172	Pyrrolidone from Proline; Carboxymethyl; 3-sulfanylpropanoyl
41	V.VPW(+44.99)PQVD(sub A)STALLGGFDLDQTC(+58.01)TLSGGK.I	Y	22.6	2907.3596	27	-2.8	970.1244	3	23.08	4610	4.71E+05	316	342	Oxidation to nitro; Carboxymethyl
42	R.E(+210.99)LTVC(sub G)NC(+58.01)PC(+58.01)VGALGAMLGGGLGRL.Q	Y	22.56	2630.1233	24	1.8	877.7166	3	23.69	5010	1.65E+05	150	173	Tri nitro benzene; Carboxymethyl
43	R.E(+21.97)LTVA(sub G)NC(+58.01)PC(+58.01)VGALGAMLGGGLGR.L	Y	22.43	2296.05	23	-1.3	766.3563	3	25.29	5333	1.77E+05	150	172	Replacement of 2 protons by magnesium; Carboxymethyl
44	L.T(+136.03)VGT(+14.02)GSR.F	Y	21.63	826.395	7	0.3	414.2049	2	13.85	1413	1.06E+04	130	136	O-Diethylphosphorylation; Methylation
45	R.V(+119.04)NGA(sub D)NTLTVGTGSRFR.D	Y	21.54	1767.9016	16	-2	590.3066	3	28.78	6462	9.61E+04	123	138	Pyridylacetyl
46	R.QC(sub W)LAH(sub L)SGGHGYSPTLR.V	Y	21.25	1682.7947	16	4.8	842.4086	2	41.03	8717	0	90	105	
47	R.E(+27.99)LT(+203.08)VGNC(+58.01)PC(+58.01)VGALGAMLGGGLGR.L	Y	21.22	2491.1394	23	3	831.3896	3	25.89	5462	1.41E+05	150	172	Formylation; HexNAcylation (ST); Carboxymethyl
48	R.V(+41.03)(+88.00)NGDNTLTVGTGSRFR.D	Y	21.13	1821.8792	16	6.8	608.3044	3	45.19	9308	0	123	138	Amidination of lysines or N-terminal amines with methyl acetimidate; 3-sulfanylpropanoyl
49	V.LLVD(sub A)Q(sub P)SIAASIAR.E	Y	20.98	1355.7772	13	7.2	678.9008	2	22	4258	1.45E+05	12	24	
50	I.N(+228.11)E(sub V)VYAGPAERGR.A	Y	20.6	1545.7535	12	2.4	516.2597	3	22.39	4390	1.68E+08	286	297	2,4-diacetamido-2,4,6-trideoxyglucopyranose
51	R.A(+88.00)INALQ(sub P)ADYSAFPHR.H	Y	20.52	1760.8303	15	1	881.4233	2	27.15	6053	8.23E+05	389	403	3-sulfanylpropanoyl
52	D.IITTAYNADREL.T	Y	20.26	1378.7092	12	8.6	690.3678	2	16.69	2354	1.02E+05	140	151	
53	R.GAD(sub G)QNL(sub F)GIVYE.A	Y	20.22	1177.5615	11	-2.4	589.7866	2	13.16	1239	3.54E+05	213	223	
54	R.E(+41.03)LTVG(+88.00)NC(+58.01)PC(+58.01)VGALGAMLGG.G	Y	20.19	2005.8617	19	3.2	669.63	3	27.98	6197	1.09E+05	150	168	Amidination of lysines or N-terminal amines with methyl acetimidate; 3-sulfanylpropanoyl; Carboxymethyl
55	R.D(sub G)H(+70.04)FDGYHAIPRNLANWS	Y	20.06	2081.9707	17	-3.9	1041.9885	2	20.19	3709	4.90E+05	485	501	Crotonaldehyde
56	H.E(-15.01)(+21.98)NPLASTTAVLIESFGER.A	Y	19.97	1939.9503	18	-1.2	647.6566	3	30.11	7389	3.26E+06	371	388	ISD (z+2)-series; Sodium adduct
57	P.ALAIATN(sub I)F(+17.99)QNDPR.T	Y	19.76	1447.7219	13	-9.5	483.5767	3	17.31	2541	1.43E+06	265	277	Fluorination
58	Y.QS(sub N)Y(+122.01)ANDDEPLSALYGFDDGR.H	Y	19.6	2353.9739	20	0.8	1177.9951	2	16.69	2413	3.47E+05	451	470	O-Isopropylphosphorylation
59	P.C(+58.01)(+162.05)VGALGAM(-4.99)LGGGLGR.L	Y	19.56	1545.757	15	0.4	516.2598	3	26.04	5578	0	158	172	Carboxymethyl; Hexose; Methionine replacement by azido homoalanine
60	R.D(+41.03)IITTAYNADR.E	Y	19.12	1292.6361	11	0.3	647.3255	2	15.67	2045	3.16E+05	139	149	Amidination of lysines or N-terminal amines with methyl acetimidate
61	R.N(+68.06)(+28.03)FAYSRVNGDNTLTVGTGSR.F	Y	19.04	2224.1235	20	-3.4	742.3793	3	20.13	3591	1.29E+07	117	136	Piperidination; Dimethylation
62	C.PC(+58.01)VGP(sub A)LGAMF(sub L)GGGLGR.L	Y	19.04	1545.7432	16	9	516.2596	3	41.13	8731	0	157	172	Carboxymethyl
63	R.TDTTGILINVVY(+44.99)AG.P	Y	18.93	1480.741	14	0.5	494.5879	3	44.19	9167	0	278	291	Oxidation to nitro

(to be continued)

(continued)

64	V.G(+71.04)ALGV(sub A)MLGGGLGR.L	Y	18.8	1227.6758	13	0.3	614.8453	2	20.67	3759	2.75E+06	160	172	Propionamide (K, X@N-term)
65	T.L(+56.06)P(sub T)VGTGSR.F	Y	18.76	841.5021	8	-0.3	421.7582	2	30.19	6773	0	129	136	Diethylation
66	R.H(+183.98)V(sub F)FANAVAIAASWTDDAAAE.A	Y	18.57	2212.937	20	0	738.653	3	19.94	3484	1.15E+04	404	423	3-Sulfobenzoic succinimidyl ester
67	G.N(-15.01)C(+58.01)PC(+58.01)VGALA(sub G)AMLGGGLGR.L	Y	18.45	1759.8055	18	-3.3	880.9071	2	32.44	7207	2.36E+05	155	172	ISD (z+2)-series; Carboxymethyl
68	N.T(+71.04)LT(+79.96)VGTGSR.F	Y	18.31	1041.4761	9	-3.9	521.7433	2	19.38	3382	8.26E+05	128	136	Propionamide (K, X@N-term); Sulfation
69	V.L(+41.03)LVAS(sub P)SIAASIAR.E	Y	18.31	1311.7874	13	7.9	438.2732	3	23.66	4866	4.88E+05	12	24	Amidination of lysines or N-terminal amines with methyl acetimidate
70	V.V(+28.03)Y(+15.99)AGPAER.G	Y	18.16	905.4606	8	0.3	453.7377	2	12.54	1134	4.91E+05	288	295	Ethylation; Oxidation or Hydroxylation
71	R.AN(sub I)AS(+159.93)LYEPLALSMPT.D	Y	18.15	1736.7134	15	4.2	579.9142	3	21.07	4008	3.72E+07	298	312	Pyrophosphorylation (ST)
72	R.EM(sub I)AS(+79.96)EVYGPEWPEFANATER.W	Y	18.12	2391.9624	20	0.1	798.3282	3	17.93	2744	1.87E+04	37	56	Sulfation
73	R.H(+28.03)(+183.04)FFANAVAIAASWTDDAA.A	Y	18.06	2087.9412	18	2.1	696.9891	3	21.74	4167	2.50E+05	404	421	Ethylation; Aminoethylbenzenesulfonylation
74	R.H(+233.05)FFP(sub A)NAVAIAASWTDD.A	Y	18.02	1993.8668	16	0	665.6296	3	29.66	6634	0	404	419	5-dimethylaminonaphthalene-1-sulfonyl
75	R.N(+43.01)C(sub F)AYSRVNGDNTLTVGTGSR.F	Y	17.96	2126.9763	20	6.2	1064.5021	2	41.13	8730	0	117	136	Carbamylation
76	A.W(sub L)GAMLGGGL(+53.97)GR.L	Y	17.7	1127.5157	11	4.8	564.7679	2	12.73	1124	2.98E+05	162	172	Trifluoroleucine
77	R.NV(sub F)AY(+15.01)SRVNGDNTLTVGTGSR.F	Y	17.69	2095.0405	20	1.2	1048.5288	2	32.97	7348	2.79E+05	117	136	Tyrosine oxidation to 2-aminotyrosine
78	K.C(+127.06)(sub R)LTALKKK.Y	Y	17.57	1030.6208	8	-6.8	516.3142	2	33.6	7395	2.14E+04	473	480	N-Succinimidyl-2-morpholine acetate
79	C.PC(+58.01)VGALV(sub G)AMM(sub L)GGGLGR.L	Y	17.51	1545.7466	16	7.6	516.2601	3	39.65	8509	0	157	172	Carboxymethyl
80	R.H(+183.04)FFAN(+28.03)AVAIAASWTDD.A	Y	17.49	1945.8668	16	3.4	649.6318	3	31.51	7075	0	404	419	Aminoethylbenzenesulfonylation; Dimethylation
81	C.L(sub P)C(+58.01)VGALGD(sub A)MLGGGLGR.L	Y	17.47	1545.7643	16	-4.1	516.2599	3	28.29	6277	0	157	172	Carboxymethyl
82	K.Y(+43.01)DS(sub P)RGHFDGYHAIPR.N	Y	17.47	1832.8342	15	-1.6	611.951	3	16.81	2385	3.69E+05	481	495	Carbamylation
83	R.V(+68.06)N(+28.03)GDNTLTVGTGSRFR.D	Y	17.32	1788.9482	16	1.9	597.3245	3	26.18	5607	3.02E+04	123	138	Piperidination; Dimethylation
84	R.E(+134.05)(+14.02)TGPSNGSVWPR.E	Y	17.22	1433.6687	12	5.7	717.8457	2	17.9	2627	2.84E+04	25	36	3-methyl-2-pyridyl isocyanate; Methylation
85	I.N(+204.00)VVY(+108.00)AGPAERGR.A	Y	17.21	1599.6642	12	7	534.2324	3	19.09	3272	1.28E+07	286	297	Addition of DFDNB crosslinker; O-Ethylphosphorylation
86	E.LT(+77.99)VGNC(+58.01)PC(+58.01)VGALGAMLGGGL GR.L	Y	17.17	2209.0095	22	-0.9	553.2592	4	25.22	5323	2.91E+05	151	172	Methylphosphonylation; Carboxymethyl
87	C.P(+148.04)C(+58.01)VGALGAMW(sub L)GGGLGR.L	Y	17.08	1706.7756	16	-3.9	854.3918	2	24.2	4969	8.87E+03	157	172	Glycosyl-L-hydroxyproline; Carboxymethyl
88	R.E(+214.97)TGP(+88.00)SNGSVWPR.E	Y	16.96	1588.5745	12	2.9	530.5336	3	16.54	2352	1.77E+06	25	36	4-sulfophenyl isothiocyanate; 3-sulfanylpropanoyl
89	R.AIL(sub N)AP(sub L)PADYSAFPHR.H	Y	16.89	1624.8361	15	4.3	813.4288	2	18.39	3000	4.33E+05	389	403	
90	V.GS(sub A)LGAMLGGGLGR.L	Y	16.89	1144.6023	13	-0.1	573.3083	2	16.57	2398	9.93E+05	160	172	
91	T.A(+298.19)Y(-2.02)NADRELTVGNC(+58.01)PC(+58.01)VGALGAMLGG GLGR.L	Y	16.76	3246.551	29	6.3	1083.1978	3	26.33	5749	1.40E+07	144	172	Levuglandinyl-lysine anhyropyrrole adduct; 2-amino-3-oxo-butanoic_acid; Carboxymethyl
92	R.QWLP(sub A)L(+53.97)SGGHGYSPTLR.V	Y	16.54	1821.8773	16	8.5	608.3049	3	26.53	5731	0	90	105	Trifluoroleucine
93	P.AL(+53.97)AIATIL(sub F)QNDPR.T	Y	16.52	1448.7599	13	7.2	483.9307	3	17.49	2796	5.04E+04	265	277	Trifluoroleucine
94	R.ET(+114.04)GP(-27.99)SNGSVWPREIAS.E	Y	16.5	1771.8489	16	9.2	591.629	3	20.56	3795	1.34E+05	25	40	Ubiquitin; Pyrrolidone from Proline
95	R.DM(sub I)ITTAYNADR.E	Y	16.44	1269.5659	11	3.2	635.7923	2	22.13	4306	7.45E+04	139	149	
96	R.E(+41.03)LT(+95.94)VGNC(+58.01)PC(+58.01)VGALGA MLGGGL.G	Y	16.44	2183.9124	21	0.1	728.9781	3	19.84	4216	4.42E+05	150	170	Amidination of lysines or N-terminal amines with methyl acetimidate; Thiophosphorylation; Carboxymethyl
97	R.VNGDNTLT(+164.06)VGTGSRFRD.I	Y	16.41	1971.9415	17	-1.4	658.3202	3	25.44	5413	4.91E+05	123	139	O-Diisopropyl-phosphorylation
98	R.V(sub E)IAS(+88.00)EVYGPEWPEFANATER.W	Y	16.28	2352.0732	20	6.5	589.0294	4	21.55	4215	8.39E+04	37	56	3-sulfanylpropanoyl
99	R.V(+42.01)ND(sub G)DNTLTVGTGSRFR.D	Y	16.24	1792.8704	16	-6.1	897.437	2	26.36	6138	1.63E+06	123	138	Acetylation (N-term)

(to be continued)

(continued)

100	T.A(+68.06)YN(+29.04)ADRELTVGNC(+58.01)PC(+58.01)VGALGAMLGGGLGR.L	Y	16.21	3047.4753	29	4.6	1016.837	3	24.01	4920	4.63E+05	144	172	Piperidination; Deamidation followed by esterification with ethanol; Carboxymethyl
101	P.C(+58.01)(+127.06)VS(sub G)ALGAMLGGGLGR.L	Y	16.17	1545.7643	15	-4.3	516.2598	3	40.54	8643	0	158	172	Carboxymethyl; N-Succinimidyl-2-morpholine acetate
102	R.H(+183.04)V(sub L)SDTGRQWLALSGGHGYS.P	Y	16.14	2209.9963	19	-2	553.5052	4	25.41	5454	4.87E+05	83	101	Aminoethylbenzenesulfonylation
103	K.YD(+43.04)A(sub P)RGHFDGYHAIPR.N	Y	16.01	1816.8757	15	5.4	606.6358	3	37.43	8179	0	481	495	Carboxyl modification with ethanolamine
104	R.V(+58.01)NGD(+15.99)NTLTVGTGSR.F	Y	16.01	1463.6852	14	-2.3	488.9012	3	36.63	8046	8.01E+03	123	136	Carboxymethyl (KW, X@N-term); Oxidation or Hydroxylation
105	R.E(+37.96)LTG(sub V)GNC(+58.01)PC(+58.01)VGALGAMLG.G	Y	15.93	1815.7244	18	5.1	606.2518	3	29.36	6626	1.06E+05	150	167	Replacement of proton by potassium; Carboxymethyl
106	L.SGGHGYSPT(+42.01)LR.V	Y	15.89	1172.5574	11	-6	587.2825	2	20.43	3693	1.02E+06	95	105	Acetylation (TSCYH)
107	A.Q(sub L)PAD(-15.99)YSAFPHR.H	Y	15.64	1271.6046	11	-3.1	424.8742	3	15.61	2140	5.44E+05	393	403	Deoxy
108	R.V(+119.04)NGD(-15.99)NTLTVGTGSR.F	Y	15.54	1492.7271	14	-0.7	498.5826	3	13.44	1368	1.54E+06	123	136	Pyridylacetyl; Deoxy
109	R.HLS(+86.00)DTGRQWLALSGGHGYS.P	Y	15.53	2126.9771	19	5.1	1064.5012	2	39.92	8551	0	83	101	Malonylation
110	V.GV(sub A)LGAM(+88.00)LGGGLGR.L	Y	15.5	1244.637	13	-1.6	415.8856	3	13.31	1296	2.57E+05	160	172	3-sulfanylpropanoyl
111	R.FRA(sub D)II(+88.00)TTAYNADRELTVGNC(+58.01)PC(+58.01)VGALGAMLGGGLGRLQGLHGL.T	Y	15.42	4559.2544	43	-1.7	912.8566	5	33.36	7464	1.60E+04	137	179	3-sulfanylpropanoyl; Carboxymethyl
112	T.LVLLVAPSIAAS.I	Y	15.38	1152.7118	12	7.1	577.3672	2	22.13	4299	9.85E+05	10	21	
113	A.ASWTDDAAAEAADAW(+162.05)AVEWR.D	Y	15.38	2353.0134	20	-0.3	589.2604	4	28.86	6446	4.00E+04	414	433	Hexose
114	R.E(+105.90)LT(+126.10)VGNC(+58.01)PC(+58.01)VGALGAMLGGGLGR.L	Y	15.26	2492.0667	23	0.1	1247.0408	2	18.12	3156	4.03E+07	150	172	Replacement of proton by silver; Octanoyl; Carboxymethyl
115	D.LSLGV(sub L)RHLSDTGR.Q	Y	15.22	1409.7739	13	-7.9	470.9282	3	33.64	7506	0	77	89	
116	R.E(+43.04)TGP(-27.99)SNGSVWPR.E	Y	15.22	1300.6523	12	-1.1	651.3327	2	15.64	1992	3.84E+05	25	36	Carboxyl modification with ethanolamine; Pyrrolidone from Proline
117	G.RQWLALSGGHGYSPTLR(+55.92).V	Y	15.18	1953.9108	17	7.1	652.3155	3	24.8	5192	2.68E+04	89	105	Replacement of 2 protons by nickel
118	A.T(+58.01)(sub S)TTAVLIESFGER.A	Y	15.14	1480.7408	13	1.1	741.3785	2	16.54	2805	7.36E+07	376	388	Carboxymethyl (KW, X@N-term)
119	Q.G(-15.01)L(+53.97)HGLTSDALR.E	Y	15.06	1177.5703	11	2.7	589.794	2	33.15	7976	1.23E+06	175	185	ISD (z+2)-series; Trifluoroleucine
120	K.K(+27.99)YDPRGHFDGYHAIPR.N	Y	15.05	1955.939	16	1.8	652.9881	3	25.65	5470	1.94E+06	480	495	Formylation
121	R.D(sub V)NGD(+37.96)NTLTVGTGSRFR.D	Y	15.05	1746.7687	16	-0.7	583.2631	3	12.16	947	2.95E+04	123	138	Replacement of proton by potassium
